# SNORA74A Drives Self‐Renewal of Liver Cancer Stem Cells and Hepatocarcinogenesis Through Activation of Notch3 Signaling

**DOI:** 10.1002/advs.202504054

**Published:** 2025-04-24

**Authors:** Ziheng Zhou, Yang Gu, Zhibin Yi, Jianyi Wang, Zhen Xiong, Hui Guo, Ying Du, Xiaoxiao Zhu, Lei He, Weizheng Ren, Yong Tian, Yanying Wang, Zusen Fan

**Affiliations:** ^1^ State Key Laboratory of RNA Science and Engineering State Key Laboratory of Epigenetic Regulation and Intervention Institute of Biophysics Chinese Academy of Sciences Beijing 100101 China; ^2^ Ministry of Education Key Laboratory of Cell Proliferation and Regulation Biology, Beijing Key Laboratory of Gene Resource and Molecular Development, College of Life Sciences Beijing Normal University Beijing 100875 China; ^3^ University of Chinese Academy of Sciences Beijing 100049 China; ^4^ Department of Hepatobiliary Surgery PLA General Hospital Beijing 100853 China

**Keywords:** DCAF13, liver cancer stem cells, notch3 signaling, self‐renewal, SNORA74A

## Abstract

Liver cancer stem cells (CSCs) account for tumor initiation, heterogeneity and therapy resistance. However, the role of small nucleolar RNAs (snoRNAs) in the regulation of liver CSCs remains largely unclear. Here, this work identifies a conserved H/ACA box snoRNA *SNORA74A* which is highly expressed in liver CSCs. *SNORA74A* deletion impaired the self‐renewal of liver CSCs and suppressed hepatocarcinogenesis. Mechanistically, highly expressed *SNORA74A* in liver CSCs bound DCAF13 to prevent K48 linked ubiquitination of E2F2 for degradation. E2F2 induced *NOTCH3* transcription to initiate Notch3 signaling activation, leading to self‐renewal of liver CSCs and hepatocarcinogenesis. Moreover, expression levels of *SNORA74A* and NOTCH3 are positively related with severity and poor prognosis of hepatocellular carcinoma (HCC) patients. Of note, antisense oligonucleotides (ASOs) against *SNORA74A* showed effective efficacy for HCC tumors, suggesting *SNORA74A* might be a potential therapeutic target for HCC therapy by eliminating liver CSCs.

## 1 Introduction

Hepatocellular carcinoma (HCC) is the most common primary liver malignancy and the third leading cause of cancer‐related deaths worldwide with a steadily increased incidence.^[^ ^1^ ^]^ Despite advances in surgical resection, systemic therapies, and immunotherapies, the prognosis of HCC remains unsatisfactory due to high recurrence rates and heterogeneity.^[^ ^2^, ^3^ ^]^ There is an urgent need to develop new therapeutic strategies based on a deeper understanding of the mechanisms underlying liver cancer. Cancer stem cells (CSCs) are considered crucial drivers of tumor heterogeneity and recurrence,^[^ ^4^ ^]^ characterized by their ability to self‐renewal capabilities, leading to tumor initiation and progression.^[^ ^5^ ^]^ Liver CSCs are currently identified and isolated using surface markers such as CD13, CD133, CD44, CD90, and EpCAM, which are well established in research.^[^ ^6^ ^]^ Although clinical therapies against these surface markers have been shown to alleviate tumor progression,^[^ ^7^, ^8^ ^]^ the potential of nucleic acid‐based therapies to provide new insights into HCC treatment remains largely unexplored. Thus investigating precise interplay among snoRNAs, a conserved class of non‐coding RNAs, in regulation of liver CSC self‐renewal mechanisms is urgently needed.

CSCs, like embryonic and tissue stem cells, rely on activation of stemness signaling pathways,^[^ ^9^ ^]^ including Notch, Hippo/YAP1, Wnt/β‐catenin, and Hedgehog pathways.^[^ ^10^ ^]^ Aberrant activation of these pathways may trigger uncontrolled cell proliferation and abnormal differentiation, leading to tumorigenesis.^[^ ^11^ ^]^ Notch signaling, a highly conserved pathway, is involved in cancer initiation, progression, immune cell responses, and tumor immunogenicity.^[^ ^12^ ^]^ Up to date, four Notch receptors (Notch 1–4) and five respective ligands have been defined.^[^ ^13^ ^]^ Ligand binding triggers proteolytic cleavage of the Notch receptor, releasing the Notch intracellular domain (NICD), which translocates to the nucleus and activates target gene transcription via recombination signal binding protein (RBP)‐Jκ, thereby initiating the transcription of Notch target genes.^[^ ^13^ ^]^ Our previously study revealed that Notch2 regulates liver CSC stemness.^[^ ^14^ ^]^ Emerging studies have revealed that RNA‐based mechanisms, including miRNAs, lncRNAs, and circular RNAs epigenetically regulate Notch signaling. However, how snoRNAs modulate Notch signaling to initiate self‐renewal in liver CSCs remains unclear.

Small nucleolar RNAs (snoRNAs) are one of the most classical categories of ncRNAs, typically 60 to 300 nucleotides in length, classified into C/D box and H/ACA box types. For their canonical functions, the C/D box snoRNAs mediate 2′‐O‐methylation of 28S ribosomal RNA (rRNA), while the H/ACA box snoRNAs facilitate pseudouridylation of 28S rRNA.^[^ ^15^ ^]^ Their nucleolar localization is directly related to their classic functions: most snoRNAs act as guide RNAs involved in the post‐transcriptional modification of rRNA and some spliceosome RNAs, while a few involved in the intranuclear processing of precursor rRNA transcripts.^[^ ^16^, ^17^ ^]^ However, increasing evidence suggests that snoRNAs can also exert non‐canonical functions to influence tumorigenesis. For example, *SNORA28* recruited BRD4 to the LIFR promoter region to activate LIFR transcription, which in turn triggered the JAK1/STAT3 pathway and enhanced the proliferation of colorectal cancer cells.^[^ ^18^ ^]^ However, the precise roles of H/ACA box snoRNAs in liver CSCs remain largely unknown. Here we identified a conserved H/ACA box snoRNA, *SNORA74A* (derived from the *MATR3* gene transcript), which was highly expressed in liver CSCs and played non‐canonical roles. *SNORA74A* depletion abrogated the self‐renewal of liver CSCs and *Snora74a* knockout impaired liver tumorigenesis. Highly expressed *SNORA74A* bound DCAF13 to prevent K48 linked ubiquitination of E2F2 for degradation. E2F2 induced *NOTCH3* transcription to initiate Notch3 signaling, enhancing the self‐renewal of liver CSCs and hepatocarcinogenesis. Administration of NOTCH3 inhibitor with ASOs against *SNORA74A* exerts synergistic therapeutic effect on HCC tumor models.

## 2 Results

### 2.1 SNORA74A is Highly Expressed in HCC Tumor Tissues and Liver CSCs

**Figure 1:**
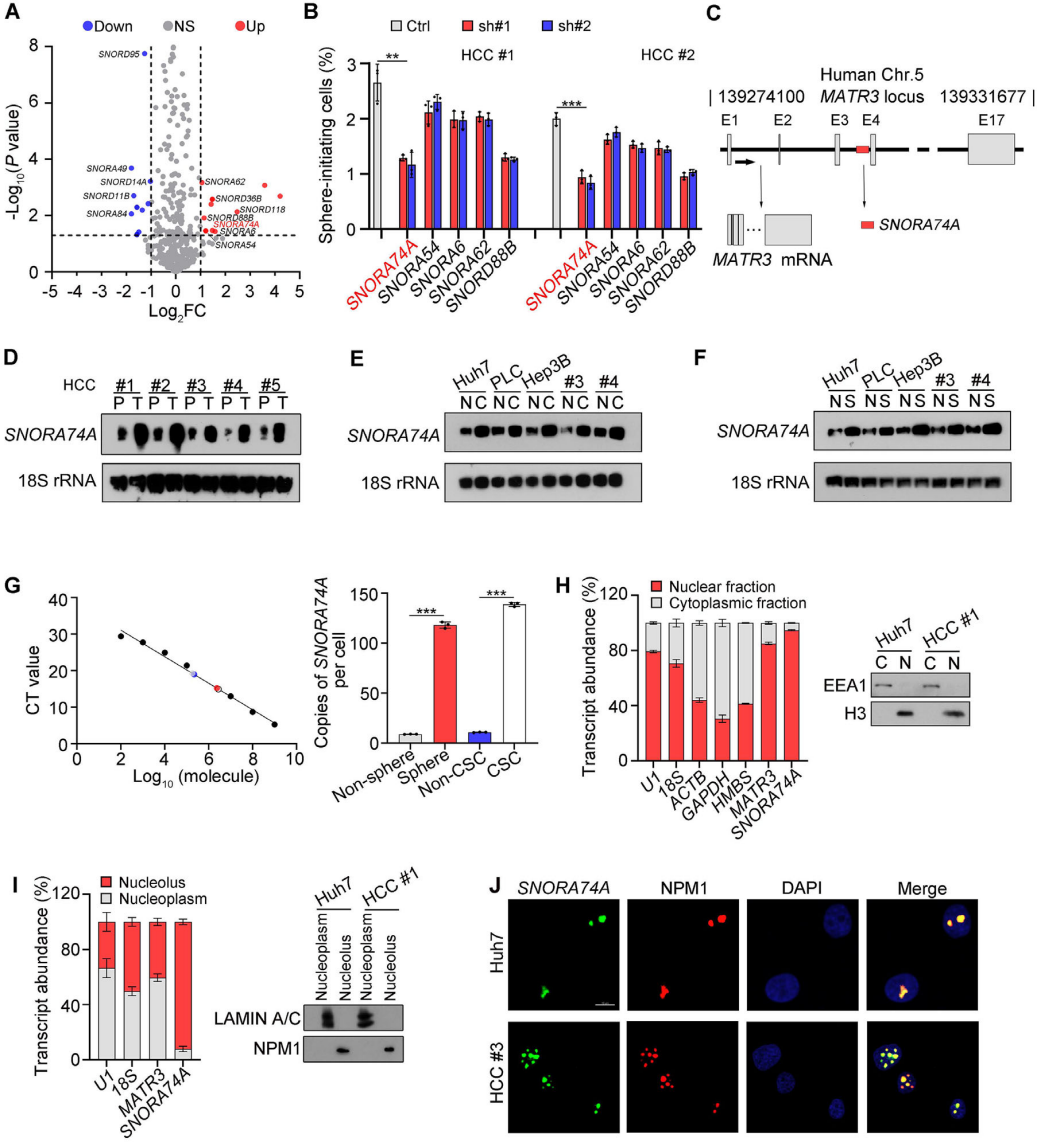
*SNORA74A* is highly expressed in HCC tumor tissues and liver CSCs. A) Volcano plot of upregulated and downregulated snoRNAs (*p* < 0.05 and fold change > 2) in liver CSCs. B) snoRNAs screening via sphere‐formation assay. Spheres with a diameter exceeding 100 µm were counted. HCC #1 and HCC #2 denote the ID of primary HCC samples. sh#1 and sh#2 indicate two effective shRNAs targeting snoRNAs. Data are presented as means ± SD. n = 3 for each group. C) Schematic representation of human *SNORA74A*. Arrow indicates the position of *SNORA74A* in the linear gene locus. *SNORA74A* marks with a red square. E1, exon #1. D–F) Expression levels of *SNORA74A* in HCC tumors and peri‐tumors (D), in CD13^+^CD133^+^ CSCs and CD13^−^CD133^−^ non‐CSCs (E), and in oncospheres and non‐spheres (F). Northern blot assays were performed to detect size and expression levels of *SNORA74A*, with 18S rRNA serving as a loading control. G) Copy numbers of *SNORA74A* was analyzed by qRT‐PCR. Left panel: black dots represent known copy numbers of *SNORA74A* from pcDNA3 plasmids containing *SNORA74A* sequence. Gray and red dots denote copy numbers of *SNORA74A* in non‐spheres and oncospheres, respectively, while blue and white dots represent copy numbers in non‐CSCs and CSCs. Right panel: Average copy numbers of *SNORA74A* per cell were calculated. Data are presented as means ± SD. n = 3 for each group. H) Nuclear‐cytoplasmic separation assays were performed using liver CSCs lysates, followed by qRT‐PCR analysis (left panel) and Western blot analysis (right panel). U1 RNA was used as a positive control for nuclear localization. EEA1, early endosome antigen 1; H3, histone 3. Data are presented as means ± SD. I) Nucleolus‐nucleoplasm separation assays were performed using liver CSCs nuclear lysates, followed by qRT‐PCR analysis (left panel) and Western blotting (right panel). LAMIN A/C, prelamin‐A/C; NPM1, nucleophosmin. Data are presented as means ± SD. n = 3 for each group. J) Representative immunofluorescence staining of *SNORA74A* and nucleolar marker NPM1 in liver CSCs sorted from Huh7 and primary HCC cells. NPM1 was used as a positive control of nucleolar staining. Scale bar, 10 µm. * p < 0.05; ** p < 0.01; *** p < 0.001 by two‐tailed Student's *t*‐test. Data are representative of at least three independent experiments.

To investigate the roles of snoRNAs in the oncogenesis of HCC, we isolated CSCs (CD13^+^CD133^+^) and non‐CSCs (CD13^−^CD133^−^) from human HCC tumor samples and conducted snoRNA transcriptome sequencing. Top differentially expressed snoRNAs were identified in liver CSCs (**Figure** **1**A). We recently revealed a C/D box snoRNA *SNORD88B* (originated from *C19orf48* gene transcript) that is involved in the modulation of liver CSCs self‐renewal.^[^ ^19^ ^]^ In this study, we focused on the role of H/ACA box snoRNAs in liver CSCs. Of the top seven high expressed snoRNAs, four H/ACA box snoRNAs and three C/D box snoRNAs were identified in liver CSCs (Figure 1A, and Figure S1A, Supporting Information). We used short hairpin RNA (shRNA) to deplete these snoRNAs in human HCC sample cells and performed oncosphere formation assays. We found that *SNORA74A* depletion showed the most significant inhibition of sphere formation compared to other candidate H/ACA box snoRNAs (Figure 1B). *SNORD88B* depletion was used as a positive control. *SNORA74A* was located in the intronic region between the third and fourth exons of the *MATR3* gene on human chromosome 5, with a length of 200 nt (Figure 1C). Based on the NCBI database, *SNORA74A* possessed three homologous transcripts, including *SNORA74B*, *SNORA74C*, and *SNORA74D*. Of these homologous transcripts, *SNORA74A* showed the highest abundance in HCC cell lines, HCC samples, spheres, and liver CSCs (Figure S1B, Supporting Information). In addition, *SNORA74A* was highly expressed in primary human HCC tumors (Figure 1D, Figure S1C, Supporting Information), liver CSCs (Figure 1E, Figure S1D, Supporting Information), and oncospheres (Figure 1F, Figure S1E, Supporting Information). However, its linear parental gene *MATR3* did not display a similar expression pattern by using qRT‐PCR (Figure S1F, Supporting Information). Furthermore, *SNORA74A* copy numbers per cell in oncospheres and liver CSCs ranged from 100 to 150 by absolute quantification measurement (Figure 1G).

Nuclear‐cytoplasmic fraction assays in liver CSCs showed that *SNORA74A* was primarily localized in the nucleus (Figure 1H). To precisely determine its nuclear localization, we further separated nucleolus and nucleoplasm and found that *SNORA74A* was predominantly localized in the nucleolus (Figure 1I), which was confirmed in liver CSCs by RNA fluorescence in situ *hybridization* (FISH) (Figure 1J). Taken together, *SNORA74A* is highly expressed in HCC tumors and liver CSCs.

### 2.2 SNORA74A Drives the Self‐Renewal of Liver CSCs and Snora74a Knockout Impairs Liver Tumorigenesis

**Figure 2:**
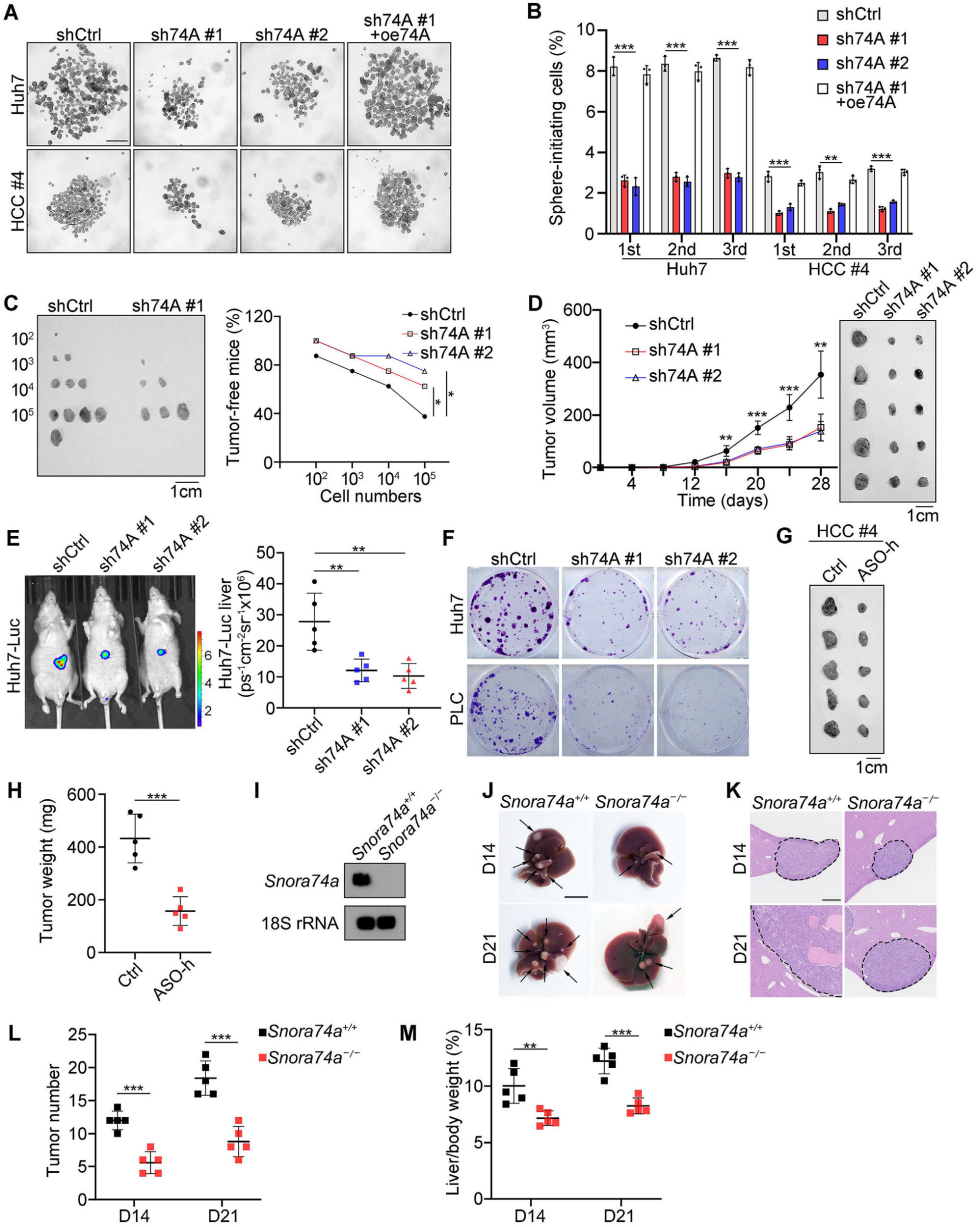
*SNORA74A* deletion inhibits self‐renewal of liver CSCs and tumorigenesis. A,B) *SNORA74A* depletion reduced oncosphere formation of Huh7 and HCC primary cells. Overexpression of *SNORA74A* (oe74A) restored oncosphere formation (A). shCtrl, shRNA scramble; sh74A, depletion of *SNORA74A*. Scale bar, 500 µm. Oncosphere formation rates were assessed through serial oncosphere formation assay (B). Data are presented as means ± SD. n = 3 for each group. C) Limited dilutions of *SNORA74A* depleted or control HCC cells were subcutaneously injected into BALB/c nude mice (8 W) and maintained for 3 months to assess tumor incidence. Representative images of tumors are shown (left panel) and numbers of tumor‐free mice were calculated (right panel). n = 8 for each group. D) 1 × 10^6^ *SNORA74A* depleted or control HCC cells were subcutaneously injected into BALB/c nude mice (8 W), followed by measurement of tumor progression every 4 days. Representative images of tumors are shown (right panel). Results are presented as means ± SD. n = 5 for each group. E) Orthotopic liver tumors of *SNORA74A* depleted or control Huh7‐Luc cells were imaged via luciferase signals. Representative images are shown (left panel), and statistical results are shown as means ± SD (right panel). n = 5 for each group. Cohen's *d* = 2.25 (sh74A #1) and 2.47 (sh74A #2). F) Representative images of clone formation capability in *SNORA74A* depleted or control HCC cells. G,H) ASO‐h (25 mg kg body weight) against *SNORA74A* was injected around HCC primary tumors in BALB/c nude mice on days 24, 26, 28, 30 and 32. Mice were sacrificed at the 40^th^ day after injection, and tumors were excised and weighed. Representative images were shown. Scale bar, 1 cm. Data are shown as means ± SD. n = 5 for each group. Cohen's *d* = 3.64. I) Northern blot analysis for *Snora74a* expression in *Snora74a* ^+/+^ and *Snora74a* ^−/−^ mouse livers, with 18S rRNA serving as a loading control. J) Liver tumors from *Snora74a* ^+/+^ and *Snora74a* ^−/−^ mice induced by hydrodynamic tail‐vein injection for 14 days (D14) and 21 days (D21). Black arrows indicate liver tumors. n = 5 for each group. Scale bars, 1 cm. K) Representative HE images of liver sections from *Snora74a* ^+/+^ and *Snora74a* ^−/−^ mice via hydrodynamic tail‐vein injection. Scale bar, 500 µm. L,M) Numbers of tumors in liver (L) and ratios of liver weight versus body weight (M) were presented after hydrodynamic tail‐vein injection. n = 5 for each group. * p < 0.05; ** p < 0.01; *** p < 0.001 by two‐tailed Student's *t*‐test. Data are representative of at least three independent experiments.

To investigate the role of *SNORA74A* in the self‐renewal of liver CSCs, we established *SNORA74A* depleted HCC cells through lentiviral‐mediated short hairpin RNA (shRNA) and confirmed the depletion efficiency using qRT‐PCR (Figure S2A, Supporting Information). The expression levels of its parent gene *MATR3* showed no significant differences (Figure S2B,C, Supporting Information). In serial sphere formation assays, we observed that depletion of *SNORA74A* significantly reduced the oncosphere formation capability of primary, second, and third‐generation spheres, while rescue of *SNORA74A* restored this ability (**Figure** **2**A,B). Flow cytometry analysis revealed that the proportion of CD13^+^CD133^+^ liver CSCs significantly decreased upon depletion of *SNORA74A* (Figure S2D, Supporting Information).

We next wanted to investigate the function of *SNORA74A* in vivo. *SNORA74A* depleted HCC cells were subjected to gradient limiting dilution and then implanted subcutaneously into immunodeficient mice. The capacity to form secondary tumors was measured to assess CSC potential, which is the gold standard for evaluating CSC potential.^[^ ^20^ ^]^ Limiting dilution analysis revealed that *SNORA74A* depletion dramatically impaired the self‐renewal capacity of liver CSCs and reduced tumor propagation (Figure 2C, and Figure S2E, Supporting Information). Additionally, we subcutaneously injected 1×10^6^ *SNORA74A* depleted or control cells into BALB/c nude mice. *SNORA74A* depleted HCC cells markedly reduced tumor propagation (Figure 2D). To assess the role of *SNORA74A* in orthotopic liver tumor development, *SNORA74A* depleted or control cells were transfected with a plasmid containing luciferase (Luc). We observed that *SNORA74A* depletion remarkably suppressed orthotopic tumor growth (Figure 2E). Clonogenic assays showed that *SNORA74A* depletion inhibited cell proliferation (Figure 2F). Of note, antisense oligonucleotides (ASOs) against *SNORA74A* were injected around HCC primary tumors dramatically inhibited tumor growth in BALB/c nude mice (Figure 2G,H), suggesting targeting *SNORA74A* might be a potential target for liver cancer therapy.

We found that *SNORA74A* was highly conserved between humans and mice (Figure S3A,B, Supporting Information). In mice, *Snora74a* was also derived from the third and fourth exons of its parental gene *Matr3* (located on chromosome 18) (Figure S3C, Supporting Information). We then constructed *Snora74a* knockout (KO) mice using CRISPR/Cas9 technology and validated the complete deletion of *Snora74a* by PCR, DNA sequencing (Figure S3D,E, Supporting Information), and Northern blot analysis (Figure 2I). Of note, *Snora74a* knockout did not affect the expression of *Matr3* gene (Figure S3F,G, Supporting Information). Moreover, *Snora74a* KO mice did not exhibit any obvious phenotypic changes and displayed normal liver development. Next, we determined the role of *Snora74a* in HCC development using a tumor induction mouse model. Hydrodynamic tail vein injection is a classic and effective model for inducing HCC in mice.^[^ ^21^, ^22^ ^]^ We injected plasmids carrying the sleeping beauty transposon system co‐expression *Hras^G12V^* and shp53 into 8‐week‐old wild type (WT) and *Snora74a* KO mice via tail vein. On days 14 and 21 post‐injection, we observed an obvious reduction in liver tumor incidence and tumor size in *Snora74a* KO mice compared to WT littermates (Figure 2J–L). Liver‐to‐body weight ratios, key indicators of tumor malignancy,^[^ ^23^ ^]^ were also significantly reduced in *Snora74a* KO mice compared to WT mice (Figure 2M). Overall, *SNORA74A* drives the self‐renewal of liver CSCs and *Snora74a* knockout abrogates tumor development and propagation.

### 2.3 *SNORA74A* Overexpression Enhances the Self‐Renewal of Liver CSCs and Tumorigenesis

**Figure 3:**
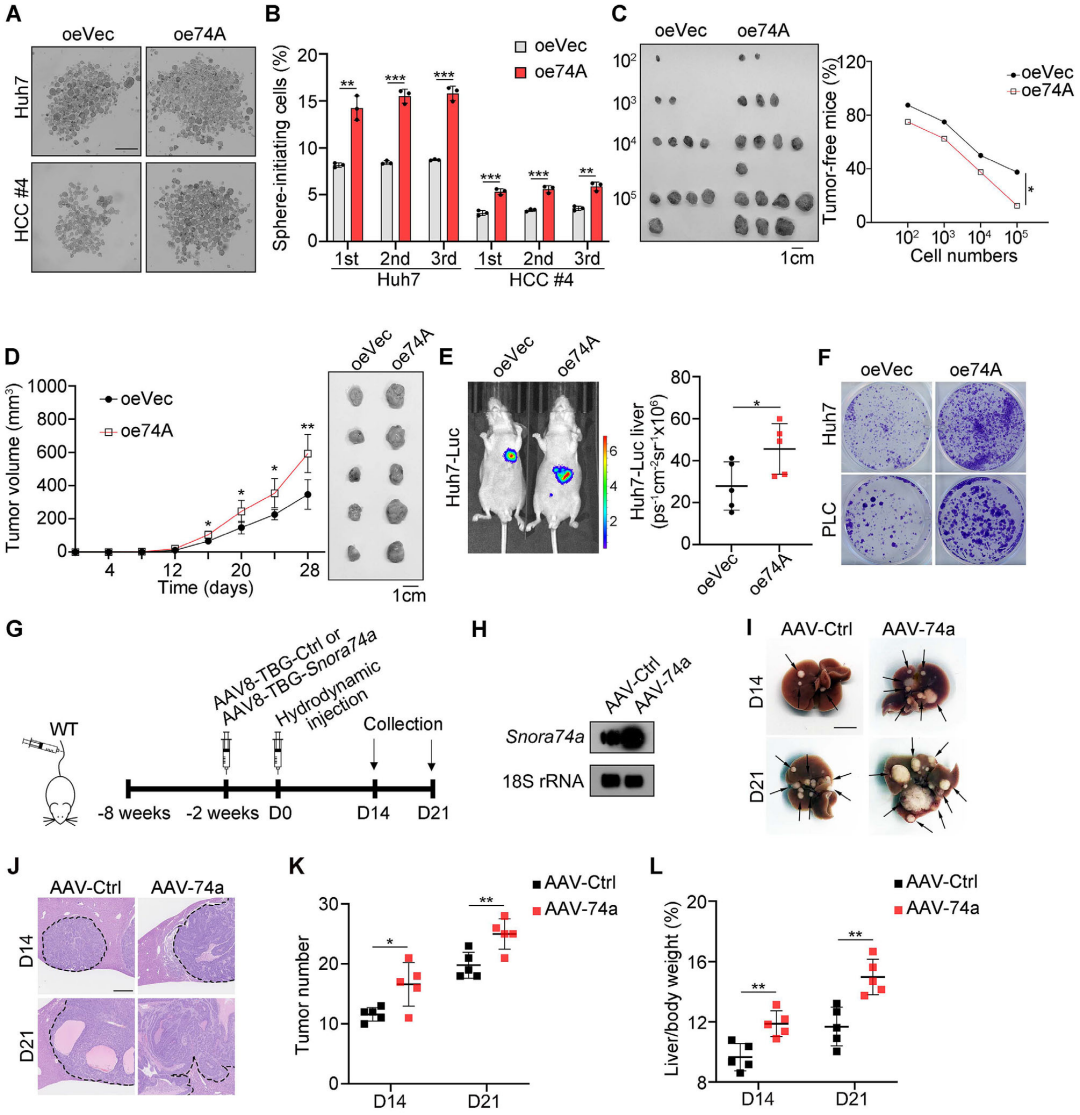
*SNORA74A* overexpression enhances self‐renewal of liver CSCs and development of HCC. A,B) *SNORA74A* overexpression enhanced oncosphere formation capability of Huh7 and HCC primary cells (A). Scale bar, 500 µm. Oncosphere formation rates were assessed through serial oncosphere formation experiments (B). Data are presented as means ± SD. n = 3 for each group. C) Limited dilutions of *SNORA74A* overexpression or control HCC cells were subcutaneously injected into BALB/c nude mice (8 W) and observed for 3 months to assess tumor incidence. Representative images of tumors are shown (left panel) and numbers of tumor‐free mice were calculated (right panel). n = 8 for each group. D) 1 × 10^6^ *SNORA74A* overexpression or control HCC cells were subcutaneously injected into BALB/c nude mice (8 W), followed by measurement of tumor progression every 4 days. Representative images of tumors are shown (right panel). Results are presented as means ± SD. n = 5 for each group. E) Orthotopic liver tumor of *SNORA74A* overexpression or control Huh7‐Luc cells were imaged via luciferase signals. Representative images are shown (left panel), and statistical results are shown as means ± SD (right panel). n = 5 for each group. Cohen's *d* = ‐1.49. F) Representative images of clone formation in *SNORA74A* overexpression or control HCC cells. G) Schematic diagram illustrating induction of liver tumors in *Snora74a* overexpression or control mice. H) Northern blot analysis for *Snora74a* expression in liver tissues from control and *Snora74a* overexpression mice, with 18S rRNA serving as a loading control. I) Liver tumor images of control and *Snora74a* overexpression mice after hydrodynamic tail‐vein injection. n = 5 for each group. Scale bars, 1 cm. J) Representative HE images of control and *Snora74a* overexpression mice liver sections after hydrodynamic tail‐vein injection. Scale bar, 500 µm. K,L) Numbers of tumors in liver (K) and ratios of liver weight versus body weight (L) were presented. n = 5 for each group. * *p* < 0.05; ** *p* < 0.01; *** *p* < 0.001 by two‐tailed Student's *t*‐test. Data are representative of at least three independent experiments.

We then overexpressed *SNORA74A* in HCC cells (Figure S2F, Supporting Information). We observed that *SNORA74A* overexpression did not affect the expression level of its parental gene *MATR3* (Figure S2G,H, Supporting Information). *SNORA74A* overexpression markedly enhanced sphere formation capability (**Figure** **3**A). Serial sphere formation assays showed that *SNORA74A* overexpression enhanced the self‐renewal capacity of liver CSCs (Figure 3B). In addition, *SNORA74A* overexpression dramatically increased proportions of CD13^+^CD133^+^ liver CSCs (Figure S2I, Supporting Information). Subsequently, limiting dilution experiments showed that *SNORA74A* overexpression obviously enhanced CSC self‐renewal capacity and increased tumor growth (Figure 3C, and Figure S2J, Supporting Information). Moreover, subcutaneous tumor formation and in vivo imaging assays revealed that *SNORA74A* overexpression remarkably promoted HCC tumor growth and propagation (Figure 3D,E). Finally, *SNORA74A* overexpression enhanced HCC cell proliferation (Figure 3F).

We next generated a mouse model with specific high expression of *Snora74a* in the liver by tail vein injection with adeno‐associated virus (AAV), driven by TBG promoter (Figure 3G). 2 weeks later, *Snora74a* was highly expressed in mouse livers by Northern blot analysis (Figure 3H). We then induced liver tumors through hydrodynamic tail vein injection in *Snora74a* overexpressed or AAV‐control mice. We observed that *Snora74a* overexpression remarkably increased both numbers and sizes of liver tumors (Figure 3I–K), as well as liver‐to‐body weight ratios in mice (Figure 3L). Collectively, *SNORA74A* overexpression promotes the self‐renewal capacity of liver CSCs and initiates tumorigenic ability.

### 2.4 *SNORA74A* Regulates Liver CSCs via a Non‐Canonical Mechanism

**Figure 4:**
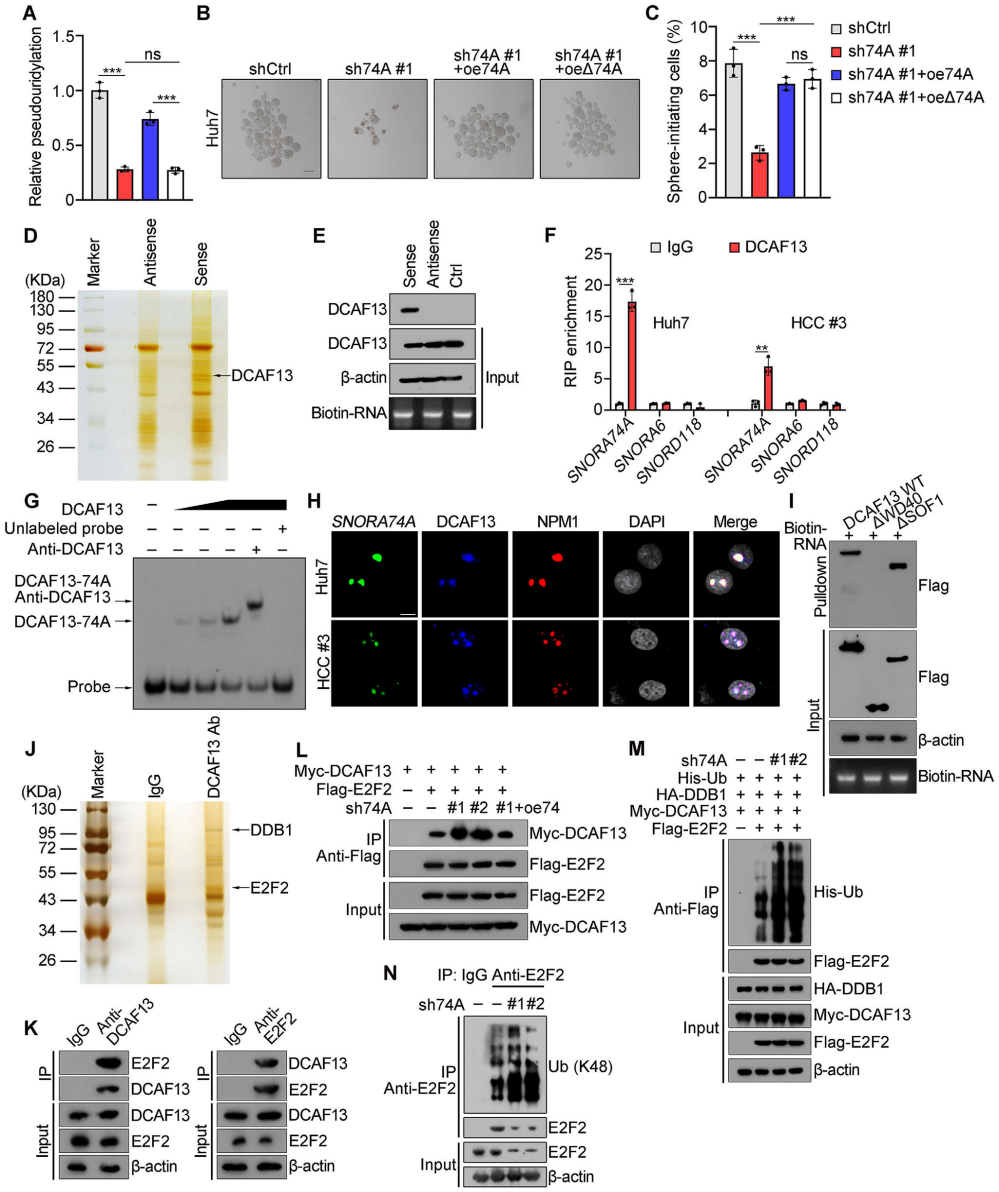
*SNORA74A* binds DCAF13 to prevent interaction with E2F2 for its ubiquitination. A) Relative pseudouridine modification levels at U3741 and U3743 of 28S rRNA in *SNORA74A* depletion, *SNORA74A* rescued, and *SNORA74A* binding region deletion (oe△74A) rescued conditions. Data are shown as means ± SD. n = 3 for each group. B,C) Oncosphere formation assays in control, *SNORA74A* depletion, *SNORA74A* rescued, or oe△74A (B). Scale bar, 100 µm. Oncosphere formation rates were assessed (C). Data are presented as means ± SD. n = 3 for each group. D) RNA pulldown assays were conducted using biotin‐labeled probes of *SNORA74A* sequence in liver CSC lysates, with its antisense sequence as a control, followed by mass spectrometry. Differential bands were confirmed to be DCAF13 (black arrow). E) Interaction between *SNORA74A* and DCAF13 was detected in liver CSCs by Western blot. β‐actin was used as a loading control. Control probes with similar length as *SNORA74A* probes but non‐specific sequences. F) Liver CSCs were used for RIP assay, followed by qRT‐PCR. *SNORA6* and *SNORD118* were used as controls. Results are shown as means ± SD. n = 3 for each group. G) Biotin‐labeled *SNORA74A* RNA probe was incubated with DCAF13 for RNA EMSA. H) Representative immunofluorescence staining of *SNORA74A*, DCAF13 and NPM1 in liver CSCs. Scale bar, 10 µm. I) Domain mapping of DCAF13 protein with a biotinylated *SNORA74A* probe, followed by RNA pulldown assay and Western blot. J) Co‐IP experiment was performed using DCAF13 and IgG antibodies in lysates of *SNORA74A* depleted liver CSCs to identify DCAF13 interacting proteins, followed by mass spectrometry. Black arrows indicate DDB1 and E2F2. K) Co‐IP experiments detected interaction between endogenous DCAF13 and E2F2 in liver CSCs. L) Myc‐tagged DCAF13 and Flag‐tagged E2F2 were co‐transfected into control, *SNORA74A* depleted or *SNORA74A* rescued liver CSCs for 48 h. Cell lysates were immunoprecipitated with anti‐Flag antibody, followed by Western blotting with anti‐Flag or anti‐Myc antibodies. M) Myc‐tagged DCAF13, Flag‐tagged E2F2, HA‐tagged DDB1, and His‐tagged ubiquitin were co‐transfected into control or *SNORA74A* depleted liver CSCs for 48 h. Cell lysates were incubated with anti‐Flag antibody for immunoprecipitation, followed by Western blotting. N) 1 × 10^6^ *SNORA74A* depleted or control liver CSCs lysates were incubated with anti‐E2F2 antibody for immunoprecipitation, followed by Western blotting with K48‐linked specific ubiquitination antibody or E2F2 antibody. * *p* < 0.05; ** *p* < 0.01; *** *p* < 0.001 by two‐tailed Student's *t*‐test. Data are representative of at least three independent experiments.

*SNORA74A* contains a H/ACA box, whose classical function mediates pseudouridylation of 28S rRNA at sites U3741 and U3743^[^ ^24^ ^]^ (Figure S4A, Supporting Information). We wanted to test whether *SNORA74A* regulated the self‐renewal of liver CSCs dependent on the pseudouridylation of 28S rRNA. We then detected pseudouridylation changes of 28S rRNA in *SNORA74A* depleted or *SNORA74A* rescued HCC cells (Figure S4B, Supporting Information). We observed that N‐cyclohexyl‐N’‐(2‐morpholinoethyl) carbodiimide methyl‐p‐toluene sulfonate (CMC) addition group in *SNORA74A* depleted HCC cells were much lower than those of control cells, indicating reduced levels of pseudouridylation modifications in *SNORA74A* depleted HCC cells (**Figures** **4**A, and S4C, Supporting Information). *SNORA74A* depletion reduced pseudouridylation modifications at U3741 and U3473 of 28S rRNA, which could be rescued by *SNORA74A* overexpression (Figures 4A, and S4C, Supporting Information). We found that deletion of *SNORA74A* matching region with 28S rRNA (12–15 nt, 181–184 nt) (Δ74A) disrupted rRNA pseudouridylation modifications. However, the deletion of *SNORA74A* pseudouridylation matching region (Δ74A) did not affect oncosphere formation (Figure 4B,C), cell proliferation (Figure S4D, Supporting Information), and expression of CSC‐related genes (Figure S4E, Supporting Information). Of note, *SNORA74A* deletion did not alter the morphology and structure of the nucleolus via transmission electron microscopy (TEM) (Figure S4F,G, Supporting Information). These results suggest that *SNORA74A*‐mediated regulation of liver CSCs self‐renewal is not dependent on its canonical mechanism.

### 2.5 *SNORA74A* Binds E3 Ligase DCAF13 to Prevent its Interaction with E2F2 for Ubiquitination

To identify associated protein candidates, we used a biotin‐labeled *SNORA74A* probe to conduct RNA pull‐down assays in liver CSCs lysates, followed by mass spectrometry analysis. DCAF13 was identified as an associated protein candidate of *SNORA74A* (Figure 4D and Figure S5A, Supporting Information). Their interaction was further validated by Western blot analysis (Figure 4E). In addition, DCAF13 could specifically precipitate *SNORA74A* in RNA immunoprecipitation (RIP) assays, but not other snoRNAs (Figure 4F). Their interaction was also confirmed by EMSA assays (Figure 4G). Immunofluorescence staining showed the co‐localization of *SNORA74A* and DCAF13 in the nucleolus of liver CSCs (Figure 4H). Domain mapping experiments revealed that the WD40 domain of DCAF13 was essential for binding to *SNORA74A* (Figure 4I, Figure S5B, Supporting Information). Deletion of the binding region with 28S rRNA did not affect the binding of *SNORA74A* to DCAF13 (Figure S5C, Supporting Information). The 1–68 nt segment of *SNORA74A* consists of an H box and a 5′‐terminal hairpin that mediates modification at U3741 site, while the 140–200 nt segment comprises an ACA Box and a 3′‐terminal hairpin that mediates modification at U3743 site. RNA truncation assay showed that these two segments of *SNORA74A* were not essential for binding DCAF13, whereas but the 69–139 nt was required for the association (Figure S5D, Supporting Information). We found that deletion of 69–139 nt did not affect rRNA pseudouridylation modifications (Figure S5E, Supporting Information), but suppressed oncosphere formation (Figure S5F, Supporting Information). These data indicate that *SNORA74A* specifically interacts with DCAF13 to regulate liver CSCs.

We observed that *SNORA74A* depletion did not alter protein levels of DCAF13 (Figure S5G, Supporting Information), suggesting that *SNORA74A* regulated liver CSCs self‐renewal in a DCAF13 protein expression‐independent manner. DCAF13 is a well‐reported E3 ubiquitin ligase that functions by mediating ubiquitination of target proteins.^[^ ^25^ ^]^ We next wanted to determine which proteins associated with DCAF13 for degradation after *SNORA74A* depletion. Using an anti‐DCAF13 antibody, we performed immunoprecipitation assays with *SNORA74A* depleted liver CSC lysates and identified E2F2 as an associated protein (Figure 4J, Figure S5H, Supporting Information). DDB1, a component of the DCAF13‐CRL4 ubiquitin ligase complex,^[^ ^26^ ^]^ was also identified (Figure 4J and Figure S5I, Supporting Information). The interaction between DCAF13 and E2F2 was further validated through co‐immunoprecipitation (co‐IP) assays (Figure 4K). Finally, we found that *SNORA74A* depletion did not alter *E2F2* mRNA levels, but reduced its protein level (Figure S5J, Supporting Information). Of note, *DCAF13* depletion caused an increase of E2F2 protein levels (Figure S5K, Supporting Information).

Similar to *SNORA74A*, the WD40 domain of DCAF13 was also the region to interact with E2F2 (Figure S6A, Supporting Information). In addition, we found that *SNORA74A* depletion enhanced the interaction between DCAF13 and E2F2, whereas *SNORA74A* overexpression reduced this interaction (Figure 4L, Figure S6B, Supporting Information). These results indicate that *SNORA74A* impairs the interaction between DCAF13 and E2F2. After cycloheximide (CHX) treatment, E2F2 was rapidly degraded in *SNORA74A* depleted HCC cells, while E2F2 in scramble treated control cells remained more stable (Figure S6C, Supporting Information). Moreover, the proteasome inhibitor MG132 treatment was able to prevent E2F2 degradation (Figure S6C, Supporting Information). Of note, *SNORA74A* overexpression prevented E2F2 degradation (Figure S6D, Supporting Information). Meanwhile, *SNORA74A* depletion promoted DCAF13‐mediated ubiquitination of E2F2 (Figure 4M), and the WD40 domain mediated ubiquitination modification of E2F2 (Figure S6E, Supporting Information). Importantly, *SNORA74A* depletion enhanced K48‐linked ubiquitination of E2F2 (Figure 4N). Finally, E2F2 protein levels were dramatically reduced in *SNORA74A* depleted liver CSCs (Figure S6F, Supporting Information), and E2F2 overexpression in *SNORA74A* depleted cells remarkably enhanced oncosphere formation (Figure S6G,H, Supporting Information). Taken together, *SNORA74A* binds DCAF13 to suppress its interaction with E2F2, preventing K48‐linked ubiquitination of E2F2 for degradation.

### 2.6 E2F2 Initiates *NOTCH3* Transcription

**Figure 5:**
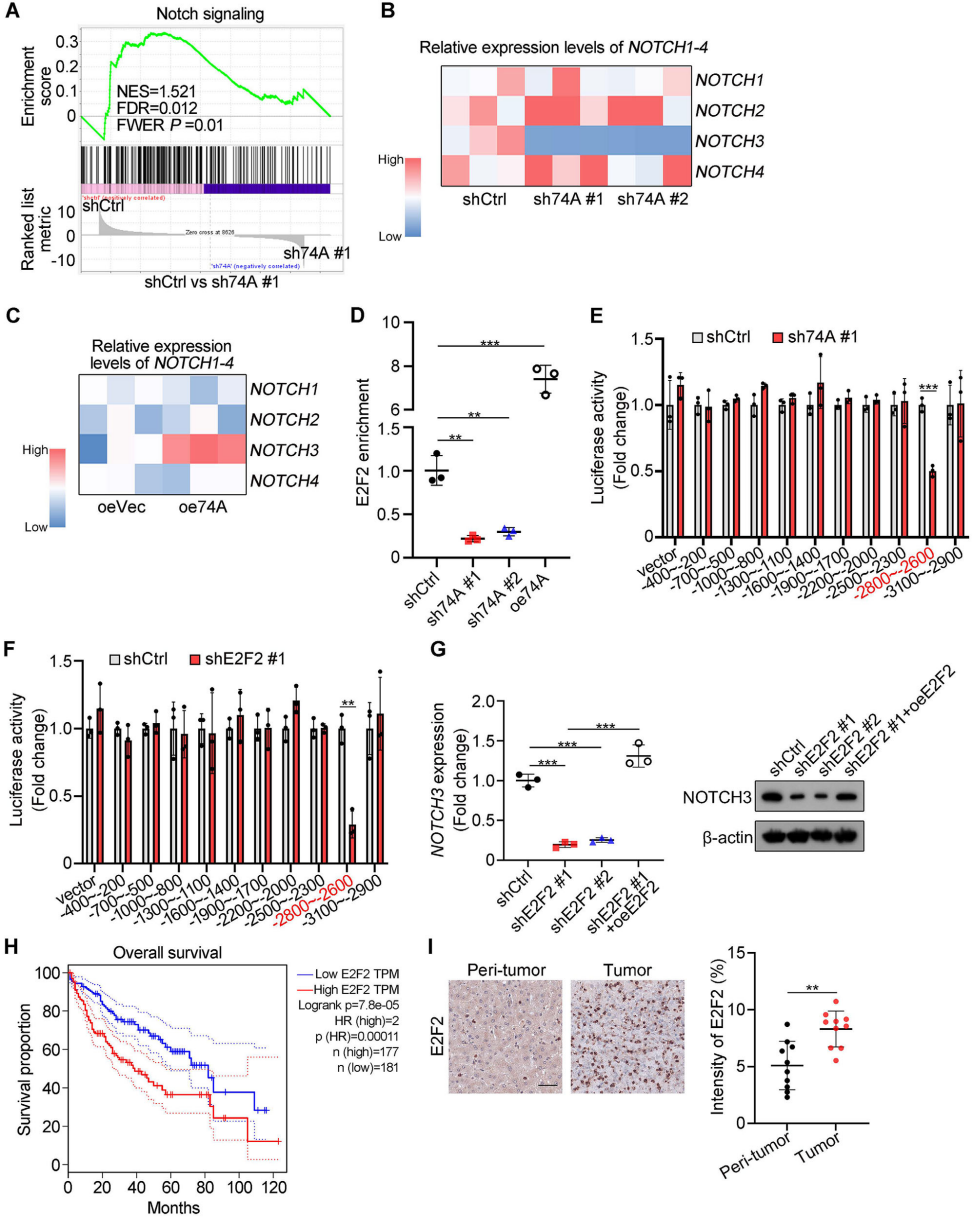
E2F2 binds to the promoter region of *NOTCH3* gene to initiate its transcription. A) GSEA analysis indicated that differentially expressed genes between *SNORA74A* depleted and control liver CSCs and Notch signaling pathway was enriched. NES, normalized enrichment score; FDR, false discovery rate; FWER, familywise error rate. B,C) Expression levels (normalized to 18S rRNA) of *NOTCH1‐4* in *SNORA74A* depleted (B) or overexpressed (C) liver CSCs were detected by qRT‐PCR. n = 3 for each group. D) ChIP‐qPCR analysis of E2F2 enrichment on the ‐2800 to ‐2600 bp region of *NOTCH3* promoter in *SNORA74A*‐overexpressed, depleted or control liver CSCs. n = 3 for each group. Results are shown as means ± SD. E) Luciferase reporter assay was performed in *SNORA74A* depleted or control liver CSCs. Results are shown as means ± SD. n = 3 for each group. F) Luciferase reporter assay was performed in *E2F2* depleted or control liver CSCs. Results are shown as means ± SD. n = 3 for each group. G) Expression levels of *NOTCH3* were assessed in control, *E2F2* depleted and *E2F2* rescued liver CSCs using qRT‐PCR (left panel) and Western blotting (right panel). Results are shown as means ± SD. n = 3 for each group. H) Kaplan‐Meier survival curves for HCC samples from GEPIA. Patients were divided into two groups based on expression levels of *E2F2*.Log‐rank *P* = 7.8E‐05 by Log‐rank (Mantel‐Cox) test I) Immunohistochemical staining of E2F2 was performed in human HCC samples (left panel). Scale bar, 50 µm. Protein expression intensity was assessed (right panel). 10 visual fields were counted using ImageJ. Data are presented as means ± SD. ** *p* < 0.01; *** *p* < 0.001 by two‐tailed Student's *t*‐test. Data are representative of at least three independent experiments.

Given that E2F2 acts as a transcription factor,^[^ ^27^ ^]^ we aimed to explore E2F2 mediated downstream target genes in the regulation of liver CSCs. We conducted transcriptome microarray analysis in *SNORA74A* depleted and control liver CSCs, followed by Gene Set Enrichment Analysis (GSEA). Among the classic stemness signaling pathways, we found that *SNORA74A* depletion only remarkably inhibited Notch signaling pathway (**Figures** **5**A, and S7A, Supporting Information). Given that the Notch receptor family contains four members, we wanted to determine which Notch receptor was mainly involved in *SNORA74A*‐mediated liver CSCs self‐renewal. We noticed that *SNORA74A* depletion only suppressed *NOTCH3* expression but no other Notch receptor members (Figure 5B). Furthermore, *SNORA74A* overexpressed only increased *NOTCH3* expression (Figure 5C). These data suggested that *NOTCH3* signaling was involved in *SNORA74A*‐mediated regulation of liver CSCs self‐renewal. We then performed chromatin immunoprecipitation (ChIP) assays using an anti‐E2F2 antibody. The 3 kb region upstream of the *NOTCH3* gene transcription start site (TSS) was mapped into 10 segments (Figure S7B, Supporting Information). We observed that E2F2 was enriched in the −2800 to −2600 bp region of the *NOTCH3* promoter (Figure S7B, Supporting Information), which was consistent with the E2F2‐binding motif predicted in the *NOTCH3* promoter by JASPAR (Figure S7C, Supporting Information). Additionally, *SNORA74A* depletion markedly decreased DNase I proteolysis sensitivity of the *NOTCH3* promoter region (Figure S7D, Supporting Information). In contrast, *SNORA74A* overexpression increased DNase I proteolysis sensitivity of the *NOTCH3* promoter region (Figure S7E, Supporting Information). Furthermore, *SNORA74A* depletion markedly reduced the enrichment of E2F2 on the *NOTCH3* promoter, whereas *SNORA74A* overexpression had the opposite effect (Figure 5D).

We next conducted a dual‐luciferase reporter assay (DLR) to verify the transcriptional activation function of E2F2 on *NOTCH3* (Figure S7F, Supporting Information). We observed that *SNORA74A* depletion reduced luciferase signals in DLR assay (Figure 5E), whereas *SNORA74A* overexpression had the opposite effect (Figure S7G, Supporting Information). We then generated *E2F2* depleted liver CSCs (Figure S7H,I, Supporting Information) and *E2F2* overexpressed liver CSCs (Figure S7J,K, Supporting Information). Of note, *E2F2* depletion reduced luciferase signals (Figure 5F), whereas *E2F2* overexpression showed an opposite effect (Figure S7L, Supporting Information). Consistently, *E2F2* depletion decreased expression levels of *NOTCH3*, while *E2F2* overexpression could restore the expression levels of *NOTCH3* compared to shCtrl cells (Figure 5G). Finally, *E2F2* expression was positively correlated with poor prognosis of HCC patients with the Cancer Genome Atlas (TCGA) dataset (Figure 5H). Immunohistochemical staining confirmed high expression of *E2F2* in HCC tumors (Figure 5I). Collectively, these results indicate that E2F2 binds to the *NOTCH3* promoter to initiate its transcription.

### 2.7 *SNORA74A* Mediated Notch3 Signaling Activation Induces Self‐Renewal of Liver CSCs and Tumorigenesis

**Figure 6:**
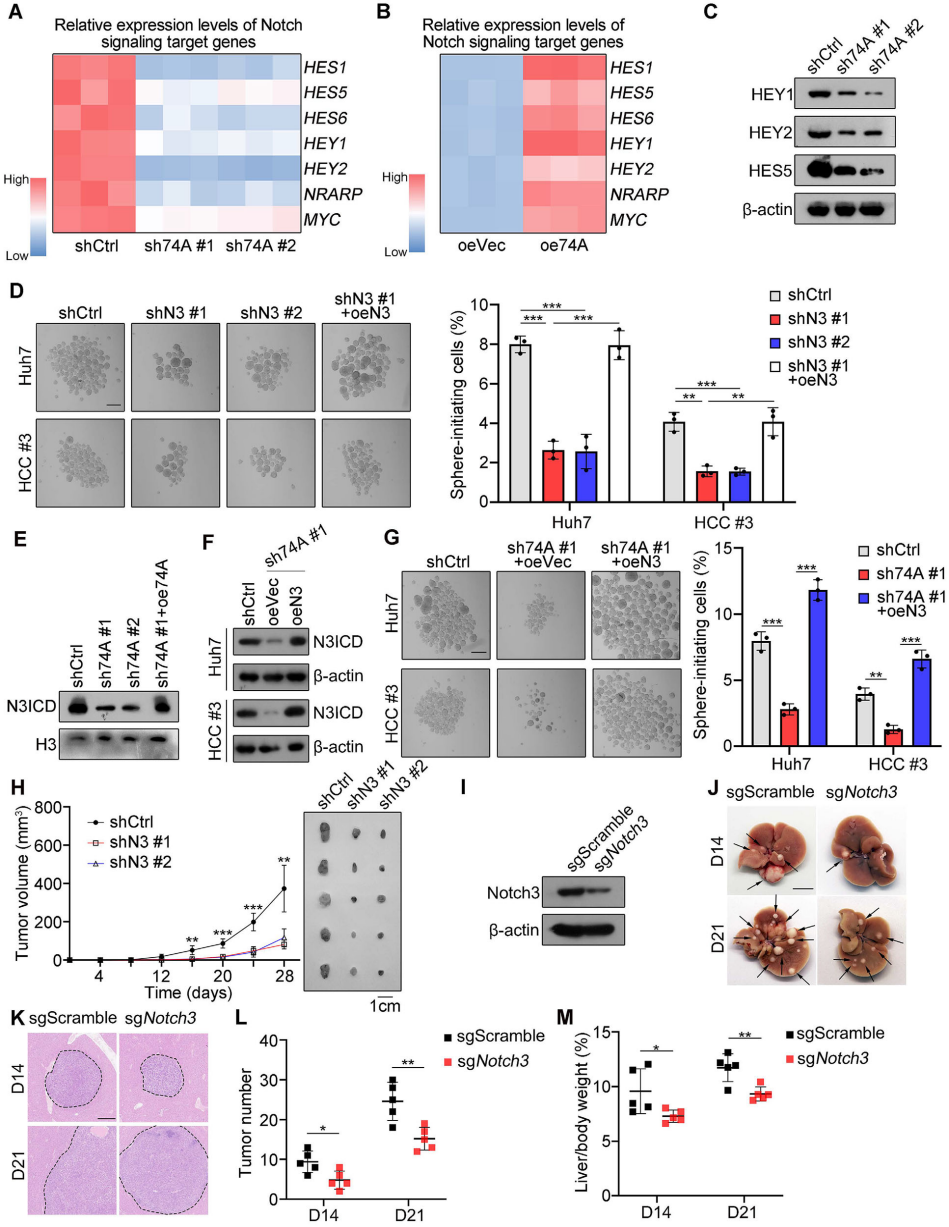
*SNORA74A* mediated Notch3 signaling initiates self‐renewal of liver CSCs and hepatocarcinogenesis. A,B) Notch signaling target genes were tested in *SNORA74A* depleted (A) or overexpressed (B) liver CSCs by qRT‐PCR. n = 3 for each group. C) Protein levels of target genes in the Notch signaling pathway were detected by Western blot with HEY1, HEY2 and HES5 antibodies in *SNORA74A* depleted and control liver CSCs. Results are shown as means ± SD. D) *NOTCH3* depletion reduced oncosphere formation capability in HCC cells. Overexpression of N3CID restored oncosphere formation that was reduced by *NOTCH3* depletion (left panel). Scale bar, 200 µm. Oncosphere formation rates were assessed (right panel). Data are presented as mean ± SD. n = 3 for each group. E) Protein levels of N3CID in the nucleus of control, *SNORA74A* depleted, and *SNORA74A* rescued liver CSCs were assessed via Western blotting, with H3 serving as a loading control. F) Western blotting was performed to assess overexpression of N3ICD in *SNORA74A* depleted cells. G) N3ICD overexpression restored oncosphere formation capability reduced by *SNORA74A* depletion (left panel). Scale bar, 200 µm. Oncosphere formation rates were assessed (right panel). Data are presented as mean ± SD. n = 3 for each group. H) 1 × 10^6^ *NOTCH3* depleted or control HCC cells were subcutaneously injected into BALB/c nude mice (8 W), followed by measurement of tumor progression every 4 days. Representative images of tumors are shown (right panel). Results are presented as means ± SD. n = 5 for each group. I) Western blotting of Notch3 expression in liver tissues from sgScramble and sg*Notch3* mice. J) Liver tumor images of sgScramble and sg*Notch3* mice after hydrodynamic tail‐vein injection for D14 and D21. n = 5 for each group. Scale bars, 1 cm. K) Representative HE images of liver sections from sgScramble and sg*Notch3* mice at D14 and D21 after hydrodynamic tail‐vein injection. Scale bar, 500 µm. L,M) Numbers of tumors in liver (L) and ratios of liver weight versus body weight (M) were presented at D14 and D21 after hydrodynamic tail‐vein injection. n = 5 for each group. * *p* < 0.05; ** *p* < 0.01; *** *p* < 0.001 by two‐tailed Student's *t*‐test. Data are representative of at least three independent experiments.

We noticed that *SNORA74A* depletion remarkably suppressed downstream target genes of Notch3 signaling (**Figure** **6**A), whereas *SNORA74A* overexpression enhanced expression of these target genes (Figure 6B). These observations were further validated by Western blot analysis (Figure 6C). We next generated *NOTCH3* depleted HCC cells and rescued the NOTCH3 intracellular domain (N3ICD) in these *NOTCH3* depleted HCC cells (Figure S8A,B, Supporting Information). We observed that *NOTCH3* depletion dramatically reduced oncosphere formation ability (Figure 6D). Of note, N3ICD overexpression in *NOTCH3* depleted HCC cells could restore oncosphere formation capacity (Figure 6D). In addition, *NOTCH3* depletion reduced proportions of CD13^+^CD133^+^ cells (Figure S8C, Supporting Information), and consequently inhibited proliferation of HCC cells (Figure S8D, Supporting Information). Importantly, depletion of *SNORA74A* in *NOTCH3* depleted cells was able to abolish oncosphere formation ability (Figure S8E,F, Supporting Information). Moreover, *SNORA74A* depletion decreased protein levels of N3ICD in the nucleus of liver CSCs (Figure 6E). N3ICD overexpression in *SNORA74A* depleted cells remarkably enhanced oncosphere formation ability (Figure 6F,G). These results indicate that *SNORA74A* regulates the stemness of liver CSCs via Notch3 mediated signaling.

To further determine in vivo function of NOTCH3 signaling, we performed subcutaneous tumor formation assays and found that *NOTCH3* depletion remarkably reduced tumor growth (Figure 6H). In addition, expression levels of E2f2 and Notch3 were decreased in HCC tumors of *Snora74a* KO mice (Figure S8G, Supporting Information). We next deleted *Notch3* in liver by tail vein injection of adeno‐associated virus into Alb‐Cre; Cas9‐KI mice (Figure 6I, Figure S8H, Supporting Information), followed with liver tumor induction by hydrodynamic tail vein injection. We noticed that *Notch3* deletion remarkably reduced both numbers and sizes of liver tumors (Figure 6J–L) and decreased liver‐to‐body weight ratios (Figure 6M). Collectively, *SNORA74A* mediated Notch3 signaling activation induces self‐renewal of liver CSCs and tumor propagation.

### 2.8 Expression Levels of *SNORA74A* and NOTCH3 are Positively Related with Severity and Poor Prognosis of HCC Patients

**Figure 7:**
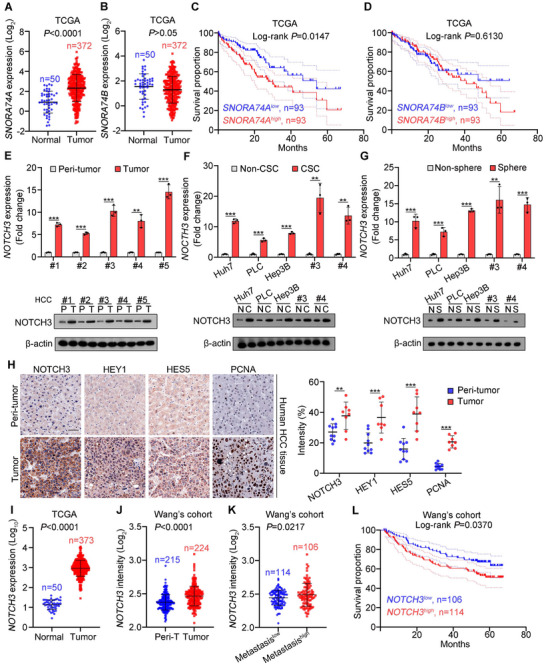
Expression levels of *SNORA74A* and NOTCH3 are positively correlated with severity of HCC patients and poor prognosis. A,B) Expression levels of *SNORA74A* (A) and *SNORA74B* (B) in HCC samples derived from TCGA. Data are presented as means ± SD. C,D) Kaplan‐Meier survival curves for HCC samples from TCGA. Patients were divided into two groups based on *SNORA74A* (C) or *SNORA74B* (D) expression levels. Log‐rank *P* by Log‐rank (Mantel‐Cox) test. E–G) Expression levels of NOTCH3 in HCC tumor and peri‐tumor tissues (E), liver CSCs and non‐CSCs (F), and oncospheres and non‐sphere cells (G) were detected by qRT‐PCR (upper panel) and Western blotting (lower panel). Results are presented as means ± SD. n = 3 for each group. H) Immunohistochemical staining of PCNA, NOTCH3, HEY1, and HES5 in human primary HCC samples (left panel). Scale bar, 50 µm. Protein expression intensities were assessed (right panel). 10 visual fields were counted using ImageJ. Data are presented as means ± SD. I–K) Expression levels of *NOTCH3* in HCC samples from TCGA dataset (I), HCC samples (J) and HCC metastatic patients (K) provided by Wang's cohort (GSE14520). Data are presented as means ± SD. L) Kaplan–Meier survival curves of HCC samples from Wang's cohort. Based on *NOTCH3* expression levels, patients were divided into two groups. Log‐rank *P* = 0.0370 by Log‐rank (Mantel‐Cox) test. ** *p* < 0.01; *** *p* < 0.001 by two‐tailed Student's *t*‐test. Data are representative of at least three independent experiments.

We next wanted to further investigate the relationship between expression levels of *SNORA74A* with NOTCH3 and HCC patient severity. We found that *SNORA74A* was highly expressed in HCC tumor tissues compared to normal liver tissues (**Figures** **7**A, and S8I, Supporting Information). In contrast, its homologous transcript *SNORA74B* showed no significant changes derived from TCGA and GSE227378 dataset (Figures 7B, and S8J, Supporting Information). Kaplan‐Meier analysis indicated that *SNORA74A* was negatively correlated with the prognosis of HCC patients (Figure 7C), whereas *SNORA74B* did not show significant correlation (Figure 7D).

We then examined expression levels of NOTCH3 in HCC tumor and peri‐tumor tissues, liver CSCs and non‐CSCs, as well as in oncospheres and non‐sphere cells. We found that *NOTCH3* expression was highly elevated in tumor tissues, liver CSCs, and spheres (Figure 7E–G). High expression of NOTCH3 was further validated by immunohistochemical staining (Figure 7H). Consistently, downstream targets of Notch3 signaling were also highly expressed in HCC samples (Figure 7H). High expression of *NOTCH3* in HCC samples was further validated by TCGA database (Figure 7I), Wang's cohort (GSE14520) (Figure 7J), and GSE227378 (Figure S8L, Supporting Information). Importantly, *NOTCH3* expression was positively associated with high metastasis in HCC patients (Figure 7K). Finally, *NOTCH3* expression was negatively related with prognosis of HCC patients (Figure 7L). Taken together, these data indicate that expression levels of *SNORA74A* and NOTCH3 are positively related with severity and poor prognosis of HCC patients.

### 2.9 NOTCH3 Inhibitor DAPT with ASOs against *SNORA74A* Manifests Synergistic Antitumor Effect on HCC Tumor Models

**Figure 8:**
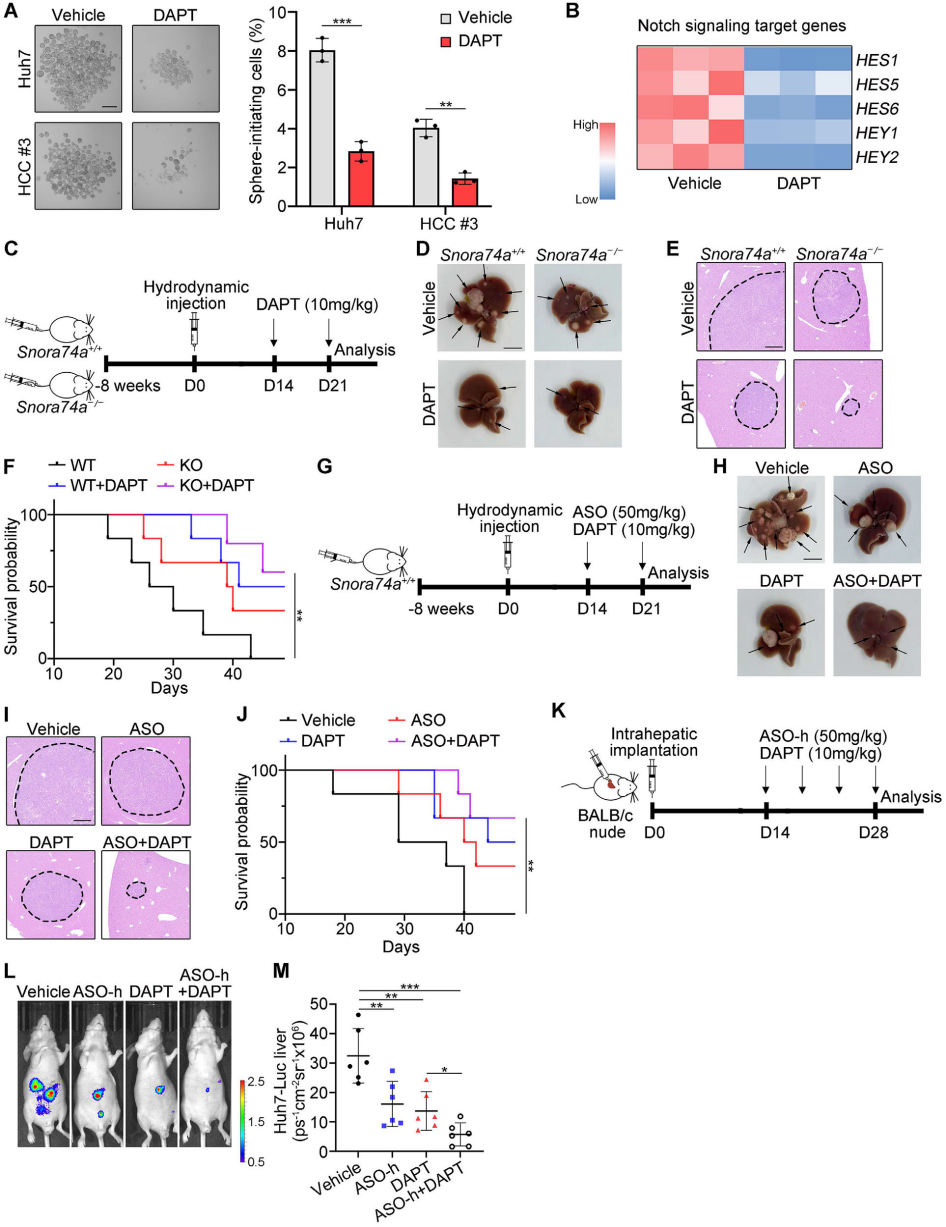
Combination of NOTCH3 inhibitor DAPT with *SNORA74A* ASOs has a synergistic antitumor effect. A) Oncosphere formation assays for DAPT treatment in HCC cells. Representative images (left panel) and statistical results (right panel) are shown. Scale bars, 500 µm. n = 3 for each group. Data are presented as means ± SD. B) Expression profiles of Notch signaling target genes with DAPT treatment. n = 3 for each group. C) Schematic representation of DAPT therapy method in *Snora74a* ^+/+^ and *Snora74a* ^−/−^ mice. D) Liver tumors from *Snora74a* ^+/+^ and *Snora74a* ^−/−^ mice treated with DAPT (10 mg kg^−1^) and vehicle at day 14 and 21. Black arrows indicate liver tumors. n = 6 for each group. Scale bar, 1 cm. E) Representative HE images of liver sections from *Snora74a* ^+/+^ and *Snora74a* ^−/−^ mice via DAPT and vehicle treatment. Scale bar, 500 µm. F) Survival curve of *Snora74a* ^+/+^ and *Snora74a* ^−/−^ mice after treatment with DAPT or vehicle. n = 6 for each group. Log‐rank *P* = 0.0042 by Log‐rank (Mantel‐Cox) test. G) Schematic representation of ASO plus DAPT treatment timeline in mice. H–J) ASOs combined with DAPT treatment in tumor‐bearing mice after hydrodynamic injection. Representative liver images (H), representative HE images (I), and survival analysis (J) are shown. n = 6 for each group. Log‐rank *P* = 0.0043 by Log‐rank (Mantel‐Cox) test. Scale bars, 1 cm. K) Schematic representation of ASO‐h/DAPT therapy method. L,M) Orthotopic human liver tumor growth was imaged via luciferase signals. Representative images are shown (L), and statistical results are shown as means ± SD (M). n = 6 for each group. Cohen's *d* = 1.93 (ASO‐h), 2.34 (DAPT), and 3.77 (ASO‐h+DAPT). * *p* < 0.05; ** *p* < 0.01; *** *p* < 0.001 by two‐tailed Student's *t* test or Log‐rank (Mantel‐Cox) test. Data are representative of at least three independent experiments.

γ‐secretase inhibitor DAPT (N‐[N‐(3,5‐diflurophenacetyl)‐L‐alanyl]‐S‐phenylglycine *t*‐butyl ester) has been used as an inhibitor of Notch3.^[^ ^28^ ^]^ We found that DAPT treatment inhibited oncosphere formation (**Figure** **8**A). Consistently, DAPT treatment also suppressed expression of Notch signaling target genes (Figure 8B). We next induced formation of HCC tumors in *Snora74a* KO and WT mice through hydrodynamic injection, followed by twice subcutaneous injection of DAPT (Figure 8C). We observed that DAPT treatment dramatically inhibited tumor formation and tumor growth (Figure 8D,E), and consequently improved survival rates of *Snora74a* KO and WT mice with bearing tumors (Figure 8F).

We next tested synergistic antitumor effect of DAPT with ASOs against *SNORA74A*. ASOs targeting *Snora74a* and ASO‐h against human *SNORA74A* were both delivered into tumors and inhibited expression of target genes (Figure S9A,B, Supporting Information). Meanwhile, no significant body, liver, spleen and kidney weight changes were detected in mice treated with ASOs or controls (Figure S9C–G, Supporting Information). Notably, administration of DAPT with ASOs showed overt synergistic antitumor effect in murine HCC tumor models (Figure 8H–J). Then, we injected human HCC‐Luc cells into livers of mice to establish orthotopic patient‐derived tumor cell (PDC) models to verify these therapeutic effects. Similarly, combined administration of DAPT with ASO‐h displayed apparent therapeutic effect on PDC models (Figure 8K–M).

Sorafenib is a drug widely used in treatment of HCC.^[^ ^29^ ^]^ In mouse HCC tumor models, administration of sorafenib with ASO against *Snora74a* exhibited remarkable synergistic antitumor effect (Figure S9H–J, Supporting Information). In parallel, administration of sorafenib with ASO‐h also displayed synergistic therapeutic effect on PDC models (Figure S9K–M, Supporting Information). Taken together, these results indicate that combined administration of DAPT with ASOs against *SNORA74A* has a synergistic therapeutic effect on HCC tumors.

## 3 Discussion

CSCs can self‐renew and differentiate, which contribute to tumor heterogeneity and resistance to conventional therapies such as chemotherapy and radiotherapy.^[^ ^30^ ^]^ In recent years, epigenetic regulation, particularly noncoding RNAs, has been defined to play an important role in the self‐renewal regulation of CSCs. Our laboratory has previously identified several key lncRNAs and circRNAs, such as *lncTCF7*,^[^ ^31^ ^]^ *lncHand2*,^[^ ^32^ ^]^ and *circIPO11* ^[^ ^33^ ^]^, which regulate the self‐renewal maintenance of liver CSCs. In our previous work, CD13 and CD133 were used as liver CSCs.^[^ ^31^ ^]^ In this study, we focused on a conserved H/ACA box snoRNA, *SNORA74A* (derived from the *MATR3* gene transcript), which was highly expressed in liver CSCs and liver tumors. *SNORA74A* depletion abrogated the self‐renewal of liver CSCs and *Snora74a* knockout impaired liver tumorigenesis. Highly expressed *SNORA74A* bound DCAF13 to prevent K48 linked ubiquitination of E2F2 for degradation. E2F2 induced *NOTCH3* transcription to initiate Notch3 signaling, leading to maintenance of the self‐renewal of liver CSCs and enhancement of hepatocarcinogenesis (Figure S10, Supporting Information). Moreover, expression levels of *SNORA74A* and NOTCH3 were positively related with severity and poor prognosis of HCC patients. Of note, ASOs against *SNORA74A* combined administration showed effective efficacy for HCC tumors, suggesting *SNORA74A* might be a potential therapeutic target for HCC therapy by eliminating liver CSCs.

In recent years, many studies have been reported that snoRNAs modulate tumor development via their canonical functions. For example, in colorectal cancer, *SNORA56* mediated pseudouridylation of 28S rRNA at site U1664 and promoted translation of the catalytic subunit of glutamate‐cysteine ligase (GCLC), thereby inhibiting ferroptosis.^[^ ^34^ ^]^ In non‐small cell lung cancer (NSCLC), *SNORD88C* enhanced 2′‐O‐methylation modification at site C3680 of 28S rRNA, which subsequently increased translation of downstream *SCD1* and promotes NSCLC proliferation and metastasis.^[^ ^35^ ^]^ In HCC, *SNORA18L5* causes hyperactive ribosome biogenesis, leading to elevated levels of mature 18S and 28S ribosomal RNAs,^[^ ^36^ ^]^ and HCC cells deficient in *SNORA24*‐guided pseudouridylation modifications increase translational misincorporation and stop codon readthrough frequencies.^[^ ^37^ ^]^ Meanwhile, increasing evidence showed snoRNAs also involved in tumorigenesis with non‐canonical mechanisms. For instance, *SNORA13* directly interacted with RPL23 to regulate the p53 pathway.^[^ ^38^ ^]^ *SNORD50A and SNORD50B* inhibited cancer progression by directly binding with K‐Ras and contributed to its degradation.^[^ ^39^ ^]^ We just reported that a C/D box snoRNA *SNORD88B* drives self‐renewal of liver CSCs through anchoring WRN in the nucleolus in a non‐canonical mechanism.^[^ ^19^ ^]^ Up to date, however, there is no report on involvement of H/ACA box snoRNA‐mediated non‐canonical mechanism in the regulation of CSCs. In this study, we demonstrated that *SNORA74A*‐mediated regulation of liver CSCs self‐renewal by binding DCAF13 to prevent K48‐linked ubiquitination of E2F2, leading to E2F2 protein stability and activation of Notch3 signaling.

Dysregulation of the Notch signaling pathway is closely related to tumor proliferation, invasion, and metastasis.^[^ ^40^ ^]^ The Notch signaling pathway is involved in the maintenance of the stem cell‐like characteristics of cancer cells, thereby enhancing cancer invasiveness.^[^ ^41^ ^]^ In this study, we demonstrated that elevated *SNORA74A* binds DCAF13 to prevent K48 linked ubiquitination of E2F2, maintaining the E2F2 stability. E2F2 is a transcription factor that is upregulated in various tumors and promotes transcription by binding to their promoter regions of target genes.^[^ ^42^, ^43^, ^44^ ^]^ We found that E2F2 binds to the −2800 to −2600 bp region of the *NOTCH3* promoter, thus enhancing its transcription. Our findings revealed that E2F2 acts as an upstream of Notch3 signaling pathway, providing the *SNORA74A*‐DCAF13‐E2F2‐NOTCH3 axis plays a critical role in the regulation of liver CSCs. Our previous study showed that C8orf4 is weakly expressed in HCC tumors and liver CSCs. C8orf4 deficiency leads to nuclear translocation of N2ICD, triggering Notch2 signaling and maintaining the stemness of liver CSCs^[^ ^14^ ^]^.

Current cancer therapies mainly target proteins, utilizing small molecules or antibodies to block or strengthen their functions. However, RNA‐targeting therapies, particularly noncoding RNAs, have been paid much attention in recent years.^[^ ^45^ ^]^ The impact of RNA‐based treatments has been recognized by the Nobel Prize in Physiology or Medicine in 2023,^[^ ^46^ ^]^ In this study, we demonstrated that ASOs against *SNORA74A* dramatically inhibit HCC tumor growth in PDC models, suggesting that targeting *SNORA74A* might be a potential strategy for treating HCC patients by eliminating liver CSCs. The high universality of RNAs enables researchers to design different structures and bioactive RNAs capable of performing specific functions.^[^ ^47^ ^]^ Targeting the AZIN1 editing site with ASOs specifically inhibits tumor incidence and growth in patient‐derived xenograft (PDX) models.^[^ ^48^ ^]^ Silencing *SNORA23* with ASOs suppresses tumor growth, dissemination of tumor cells, and liver metastasis.^[^ ^49^ ^]^

In summary, our study reveals a non‐canonical function of H/ACA box snoRNA *SNORA74A* in the regulation of liver CSCs. We showed that the *SNORA74A*‐DCAF13‐E2F2‐NOTCH3 axis initiates the self‐renewal of liver CSCs and enhances HCC tumorigenesis. Our findings suggest that snoRNAs may be potential targets for HCC therapy, paving the way to develop more effective therapeutic strategies for HCC patients by elimination of liver CSCs.

## 4 Experimental Section

### Cell Lines and HCC Samples

Human HCC cell lines Huh7, PLC, and Hep3B were provided by Dr Zeguang Han (the Shanghai Jiaotong University School of Medicine, Shanghai, China). HCC cell lines and Human 293T cells (ATCC, CRL‐3216) were maintained in DMEM supplemented with 10% FBS, 100 µg mL^−1^ penicillin and 100 U mL^−1^ streptomycin.

Primary human HCC samples were obtained from the Department of Hepatobiliary Surgery, PLA General Hospital (Beijing, China), with the approval of the Research Ethics Committee at the hospital. Clinical characteristics of HCC patients are shown in the Table S7, Supporting Information. Primary HCC cells were isolated from HCC samples, as follows. HCC tumor tissues were cut into 1 mm^[3]^ pieces with scissors in digestion buffer (0.1% IV collagenase, 0.01% DNase, 0.05% protease) at 37 °C for 45 min with agitation (1–2 times per second). Then, supernatants were collected and passed through a 100 µm cell strainer. Supernatants were centrifuged at 50 g for 1 min to collect the supernatants, then the supernatants were centrifuged at 150 g for 8 min to enrich cells in the pellet. After removing red blood cells, we obtained primary HCC cells.

### Sphere Formation Assay

Serial sphere formation assays were performed with 4000 HCC cells and a sphere formation assay was performed with 2000 HCC cells. HCC cell lines or primary HCC cells were plated in low‐attachment 6‐well plates (Corning) and cultured in serum‐free DMEM/F12 medium supplemented with 20 ng mL^−1^ bFGF, 20 ng mL^−1^ EGF, N2, and B27. Sphere formation efficiency = (number of spheres formed) / number of cells plated × 100%.

### Generation of *Snora74a* Knockout or Overexpressed Mice

*Snora74a* knockout mice on a C57BL/6J background were generated using a CRISPR‐mediated approach as described previously.^[^ ^50^ ^]^ Zygotes from C57BL/6 mice were injected with a pair of sgRNAs targeting intronic sequences flanking the *Snora74a* gene, and then transferred into the uterus of pseudo‐pregnant females to obtain F0 mice. To generate sgNotch3 mice, sgRNAs targeting the *Notch3* gene were synthesized and cloned into AAV8 delivery vectors (Catalog number 60 231, Addgene), which were then injected via tail vein into Alb‐Cre; Cas9‐KI mice for viral infection. Genomic DNA deletions were identified through PCR screening and DNA sequencing, followed by confirmation using Northern blotting. Alb‐Cre mice were purchased from GemPharmatech Co., Ltd, and Cas9‐KI mice were obtained from Jackson Laboratory. To generate *Snora74a* overexpressed mice, the *Snora74a* gene was cloned into the AAV8‐TBG‐GFP (the U6 promoter was replaced with the liver‐specific TGB promoter in the AAV8 plasmid) adenoviral vector and co‐transfected with packaging plasmids pHelper and pAnc into 293T cells. After 2 days, adenovirus was collected using a Viral Concentration Kit (C2901S, Beyotime), dissolved in 1 mL of physiological saline, and injected into mice via the tail vein using an insulin syringe at a volume of 200 µL per mouse. 2 weeks later, expression of the target gene reached its peak.^[^ ^51^ ^]^ Northern blotting was performed to detect expression levels. BALB/c nude mice and C57BL/6 mice were obtained from Beijing Vital River Laboratory Animal Technology (Beijing, China). All mice were maintained in pathogen‐free conditions under standard 12 h light‐dark cycle, fed stand rodent chow and water. All mouse experiments were conducted in accordance with the relevant guidelines and approved by the Institutional Animal Care and Use Committees (approval number: SYXK2022148) at the Institute of Biophysics, Chinese Academy of Sciences.

### Quantitative RT‐PCR

Total RNA was extracted from the respective samples using the standard Trizol instruction. Complementary DNA (cDNA) templates were synthesized using 5X All‐In‐One RT Master Mix (ABM, China). Following the manufacturer's instructions, we performed quantitative real‐time PCR analysis using the SYBR Green reaction system on the CFX Connect real‐time PCR detection system (Bio‐Rad, USA). The experiment was performed at least three times independently and the results were analyzed by 2^−ΔΔCt^ method. 18S rRNA was used as an internal reference control.

### shRNA Knockdown System

All shRNAs were designed using RNAi Designer (Thermo). Three shRNAs targeting each designated gene were selected and cloned into the pSicoR‐Puro lentiviral vector (Catalog No. 12084, Addgene). The pSicoR‐Puro vector was co‐transfected with packaging plasmids pVSVg (Catalog No. 8454, Addgene) and psPAX2 (Catalog No. 12260, Addgene) into 293T cells. Lentivirus was collected 2 days post‐transfection and filtered through a 0.45 µm sieve. After mixing with an equal volume of fresh DMEM, HCC cells were infected at 37 °C, 5% CO_2_ incubator for 24 h, followed by puromycin selection. Subsequently, HCC cells were passaged, and gene silencing efficiency was analyzed using qRT‐PCR.

### Measurement of *SNORA74A* Copy Numbers

The plasmid pcDNA3‐SNORA74A was serially diluted to generate a standard curve for *SNORA74A* quantification by qRT‐PCR analysis. The copy number of diluted pcDNA3‐*SNORA74A* was calculated using the DNA/RNA copy number calculator on the website (http://endmemo.com/bio/dnacopynum.php). To measure the copy number of *SNORA74A* in spheres versus non‐spheres, as well as in CSCs versus non‐CSCs, total RNA was extracted from 3 × 10^5^ HCC cells, reverse transcribed into cDNA, and subsequently analyzed by qPCR. Copy numbers were determined using the standard curve.

### Isolation of Cytoplasm, Nucleoplasm and Nucleolus

2 × 10^7^ HCC cells were collected and suspended in 200 µL lysis buffer (10 mM Tris pH 7.4, 140 mM NaCl, 1.5 mM MgCl_2_, 1× protease inhibitor cocktail). After incubation on ice for 10 min, the suspension was centrifuged at 300 g for 5 min at 4 °C to pellet the nuclear fraction, with the supernatant representing the cytoplasmic fraction. The nuclear pellet was further separated into nucleoplasm and nucleolus fractions; the nuclear pellet was resuspended in 20 µL sucrose solution (340 mM, 5 mM MgCl_2_) and sonicated until complete nuclei were no longer visible under a microscope. 200 µL of sucrose solution (880 mM, 5 mM MgCl_2_) was gently layered at the bottom of the sonicated nuclear fraction, followed by centrifugation at 500 g for 20 min at 4 °C to pellet the nucleoli, with the supernatant as the nucleoplasm. The nucleolar pellet was resuspended in 200 µL of 340 mM sucrose buffer for further analysis as the nucleolus fraction.

### Immunofluorescence Staining

HCC cells were fixed with 4% paraformaldehyde (PFA) for 20 min, followed by permeabilization with 1% Triton X‐100 for 30 min, prehybridized with hybridization buffer (50% formamide, 5× SSC, 500 ng µL^−1^ yeast tRNA, 1× Dehardt's solution, 500 ng µL^−1^ sperm DNA, 50 ng µL^−1^ Heparin, 2.5 mM EDTA, 0.1% Tween‐20) for 1 h at 45 °C, incubated with biotinylated *SNORA74A* probes at 45 °C for 2 h, and then washed three times with SSC washing buffer. Fluorescein TSA kit was used to couple 488 fluorescent signals to biotin. Blocking was performed with 10% donkey serum for 30 min before incubation with primary antibodies at 37 °C for 2 h. After washing with PBS, cells were then incubated with fluorescently labeled secondary antibodies at 37 °C for 1 h. After mounted with antifade reagent, images were taken using a confocal microscope (Nikon A1R+, Japan).

### Flow Cytometry

Human liver cells were stained with FITC‐conjugated CD13 and PE‐conjugated CD133 antibodies, followed by sorting using flow cytometry. Subsequent analysis was performed using FlowJo 10.8.1 software.

### PDC Models and ASOs Treatment

For tumor initiation analysis, primary HCC cells obtained from patients were subjected to serial dilutions (10^2^, 10^3^, 10^4^ and 10^5^), and then they were mixed with 100 µL of Matrigel before subcutaneous injection into BALB/c nude mice. The percentage of tumor‐free mice was calculated after 3 months. Eight parallel groups were used for each sample. For tumor formation analysis, xenografts were generated by subcutaneous injection of 1 × 10^6^ primary HCC cells into BALB/c nude mice. Tumor size was measured every 4 days. At least 5 mice were used for each group. ASO was injected at 25 mg kg^−1^ body weight around the tumor every 2 days (days 24, 26, 28, 30, and 32). Mice were sacrificed on day 40 after injection of HCC primary cells or when they developed tumors larger than 15 mm in diameter or skin ulceration. ASO sequence is shown in the Table S5, Supporting Information, which has been reported to be effective.^[^ ^52^ ^]^

### In Vivo Imaging System

1×10^6^ luciferase‐expressing Huh7 cells were orthotopically injected into the livers of BALB/c nude mice. 2 weeks later, 3 mg D‐luciferin potassium salt was injected intraperitoneally into the mice and waited for 10 min. The mice were then anesthetized with isoflurane. Luciferase activity was detected using the IVIS lumina3 machine (PerkinElmer). Results were analyzed using Living Image 4.3 software (PerkinElmer).

### Cell Proliferation Assay

5000 cells were transplanted into each well of a 6‐well plate and cultured in a cell incubator until the cells grew to 75% confluence. After washing with PBS, the cells were fixed with 4% PFA for 10 min and stained with 0.1% crystal violet at 37 °C for 30 min. The plates were gently washed with distilled water and then left to air dry in an oven.

### Hydrodynamic Tail Vein Injection

To deliver the intrahepatic transposon system, experiments were conducted using 8‐week‐old mice, which were hydrodynamically injected through the tail vein with the following plasmids: 20 µg of pBABE‐c‐mycT58A+HRasG12 V plasmid (catalog number 11 130, Addgene), 20 µg of pT2‐shP53 plasmid (catalog number 124 261, Addgene), and 20 µg of a plasmid encoding firefly luciferase and SB transposase (catalog number 20 207, Addgene). All plasmids were suspended in sterile‐filtered 0.9% NaCl solution. The total volume of the injection solution was 10% of the mouse's body weight (in mL) and was administered within less than 5 s.

### Assay of Pseudouridylation on 28S‐U3741 and U3743

Following previous methods,^[^ ^53^, ^54^ ^]^ pseudouridylation on 28S‐U3741 and U3743 was analyzed. RNA was treated with CMC, followed by reverse transcription using RNase H minus Moloney leukemia virus reverse transcriptase, RNasin ribonuclease inhibitor, and 1 mM of reverse primer targeting the sequence downstream to 28S‐U3741 and U3743, with 10 µm dNTPs in the reaction. The reaction was incubated at 37 °C for 5 min and terminated at 70 °C for 15 min. cDNA amplification was measured by qPCR, and pseudouridylation levels were calculated using the formula 2 ^(CT+CMC‐CT‐CMC)^.

### RNA Pulldown and Mass Spectrometry

Liver CSCs were lysed, and the supernatant was incubated with biotin‐labeled probes specific to *SNORA74A* or its antisense control probe. Subsequently, precipitated components were separated via SDS‐PAGE and silver stained. Differential bands enriched by *SNORA74A* were analyzed both by mass spectrometry and Western blotting.

### RNA EMSA Assay

EMSA experiments were conducted using the Chemiluminescent RNA EMSA Kit (Beyotime). Recombinant DCAF13 protein was purified (constructed into plasmids pGEX‐6P‐1 and 3xFlag). RNA fragments of *SNORA74A* were transcribed via T7 and biotin‐labeled according to standard protocols. Probes and recombinant proteins were incubated in binding buffer, and mobility shift assays were performed using native gel electrophoresis.

### Immunoprecipitation Assay

HEK293T cells or liver CSCs were transfected with the indicated plasmids and cultured for 48 h. For endogenous immunoprecipitation, 1 × 10^6^ liver CSCs were sorted for expansion. Cells were lysed in radioimmunoprecipitation assay buffer for 1 h at 4 °C. The lysates were then incubated with specific antibodies for 2 h, followed by immunoprecipitation with Protein A/G agarose beads for 1 h, and subsequently separated by SDS‐PAGE and analyzed by Western blotting.

### SnoRNA and Transcriptome Sequencing

Total RNAs isolated with Trizol regent from liver CSCs (CD133^+^CD13^+^) and non‐CSCs (CD133^−^CD13^−^) from human HCC samples were sequenced using human snoRNA Array (YINGBIO Tech, China). To identify downstream targets of *SNORA74A*, total RNAs were isolated from *SNORA74A* knockdown and control liver CSCs, followed by NimbleGen sequencing analysis (BGI Tech Company). Raw data was filtered with SOAPnuke (v1.6.5) by removing reads containing adapters (adapter contamination), removing reads whose unknown base (“N” base) ratio is more than 1%, and removing reads whose low‐quality base ratio (Base quality less than or equal to 15) is more than 40%, afterward clean reads were obtained. Between‐group differential gene analysis was performed using DEseq under conditions of Fold Change ≥ 2 and *p* value ≤ 0.001. PoissonDis was performed between‐sample differential gene analysis under conditions of Fold Change ≥ 2 and FDR ≤ 0.001.

### Chromatin Immunoprecipitation Assay

Liver CSCs were cross‐linked with 1% formaldehyde at 37 °C for 10 min. Cells were then washed twice with PBS, lysed in SDS lysis buffer (1% SDS, 10 mM EDTA, 50 mM Tris), and sonicated to generate DNA fragments of 200 to 500 bp. The lysate was pre‐cleared with protein A Agarose/Salmon Sperm DNA (50% slurry) and then incubated overnight at 4 °C with specified antibodies (4 µg each). After washing, DNA was eluted and purified from the beads. DNA fragments were analyzed using primers listed in Table S6, Supporting Information.

### Luciferase Reporter Assay

The truncated NOTCH3 promoter was cloned into the pGL3 vector for luciferase reporter analysis. Cells were seeded in 24‐well plates the day before transfection. Transfection for each well included 100 ng pGL3 plasmid and 1 ng pRL‐TK plasmid. Cells were transferred to 96‐well assay plate at 10 000 well^−1^ 24 h later. After cells were attached, luminescence signals were analyzed using the ONE‐Glo Luciferase Assay System (Promega, Madison) according to the manufacturer's protocol.

### Overexpression of *SNOA74A*, *E2F2*, and N3ICD

For *SNORA74A* and *E2F2* overexpression, their full‐length sequences were cloned into the pBPLV‐GFP vector. For N3ICD overexpression, according to the previously published method,^[^ ^55^ ^]^ *NOTCH3* CDS (AA 1662–2321) sequences were cloned into the pBPLV‐GFP vector. The pBPLV‐GFP vector was co‐transfected with packaging plasmids (pBPLV, VSVG, pLp1, and pLp2) into 293T cells. Lentivirus was collected 2 days post‐transfection and filtered through a 0.45 µm sieve. After mixing with an equal volume of fresh DMEM, HCC cells were infected at 37 °C for 24 h, followed by GFP sorting.

### DNase I Sensibility Assay

Cell nuclei were isolated according to the protocol from the nuclei‐isolation kit (catalog number 78 833, Thermo). The isolated nuclei were then suspended in 200 µL of DNase Digestion Buffer and digested with 2 U of DNase I at 37 °C for 5 min. After the digestion was stopped, total DNA was extracted and subsequently analyzed by qRT‐PCR.

### Immunohistochemical Staining

The paraffin‐embedded sections of tumor tissue were deparaffinized in xylene and dehydrated through a series of ethanol gradients. Endogenous peroxidase activity was blocked by treatment with 3% hydrogen peroxide (H_2_O_2_) for 10 min. Sections were subjected to antigen retrieval by boiling in Tris‐EDTA buffer for 20 min, followed by blocking with 10% donkey serum for 30 min at room temperature. Subsequently, the slides were incubated with primary antibodies at room temperature for 2 h. After incubation with HRP‐conjugated secondary antibodies at room temperature for 1 h, DAB staining was performed followed by counterstaining with hematoxylin, and then dehydration and mounting.

### Statistical Analysis

Data were statistically analyzed using GraphPad Prism 9.0 or Excel 2019 software with two‐tailed Student's *t‐*tests and Log‐rank (Mantel‐Cox) test. *p* < 0.05 was considered significant (* *p* < 0.05; ** *p* < 0.01; *** *p* < 0.001); ns, not significant. Each experiment was performed at least three times independently. Representative experiments were shown in respective figures. Tumorigenic cell frequency was calculated according to extreme limiting dilution analysis website instructions (ELDA, https://bioinf.wehi.edu.au/software/elda/).

## Conflict of Interest

The authors declare no conflict of interest.

## Author Contributions

Z.Z., Y.G., and Z.Y. contributed equally to this work. Z.Z., Y.G. and Z.Y. are the co‐first authors. Z.Z. designed the project, performed experiments, analyzed data and wrote the paper; Y.G. and Z.Y. performed *SNORA74A*‐related experiments; J.W. generated cell lines and identified CRISPR/Cas9‐mediated mouse models; Z. X. helped with experimental design; H.G. and Y.D. provided experimental resources and technical support; X.Z. generated animal models; L.H. and W.R. provided human HCC samples; Y.T. generated animal models and analyzed data; Y.W. designed the study and performed some experiments; Z.F. initiated, organized, designed, and wrote the paper.

## Supporting information



Supporting Information

## Data Availability

The data that support the findings of this study are available in the supplementary material of this article.
