# Supplementary material for: SNORA74A Drives Self‐Renewal of Liver Cancer Stem Cells and Hepatocarcinogenesis Through Activation of Notch3 Signaling
Source: Adv Sci (Weinh). 2025 Apr 24;12(26):2504054. doi: 10.1002/advs.202504054 (PMC12245033; doi:10.1002/advs.202504054)
Supplement: Supplementary file 1 — Supporting Information [file ADVS-12-2504054-s001.docx]

**Appendix for:**

***SNORA74A* drives self-renewal of liver cancer stem cells and hepatocarcinogenesis through activation of Notch3 signaling**

Ziheng Zhou, Yang Gu, Zhibin Yi, Jianyi Wang, Zhen Xiong, Hui Guo, Ying Du, Xiaoxiao Zhu, Lei He, Weizheng Ren, Yong Tian, Yanying Wang, Zusen Fan

**Supplementary Figures**

Figure S1. *SNORA74A* is highly upregulated in human HCC tumor tissues and liver CSCs.

Figure S2. Generation of *SNORA74A* depleted and overexpressed HCC cells.

Figure S3. Generation of *Snora74a* knockout mice.

Figure S4. *SNORA74A* drives self-renewal of liver CSCs in a canonical function independent manner.

Figure S5. *SNORA74A* regulates self-renewal of liver CSCs by interacting with DCAF13.

Figure S6. *SNORA74A* reduces ubiquitination levels of E2F2 by inhibiting the interaction between E2F2 and DCAF13.

Figure S7. E2F2 binds to the promoter region of *NOTCH3* gene.

Figure S8. *NOTCH3* promotes self-renewal of liver CSCs.

Figure S9. DAPT with ASOs against *SNORA74A* shows a synergistic anti-tumor effect.

Figure S10. Graphic abstract of this study.

**Supplementary Tables**

Table S1. Materials used in this study.

Table S2. shRNA sequences used in this study.

Table S3. sgRNAs for mouse knockout construction and primers for genotyping.

Table S4. qPCR primers used in this study.

Table S5. ASO against *SNORA74A* used in this study.

Table S6. Primers for ChIP assay in this study.

Table S7. Clinical characteristics of HCC patients.


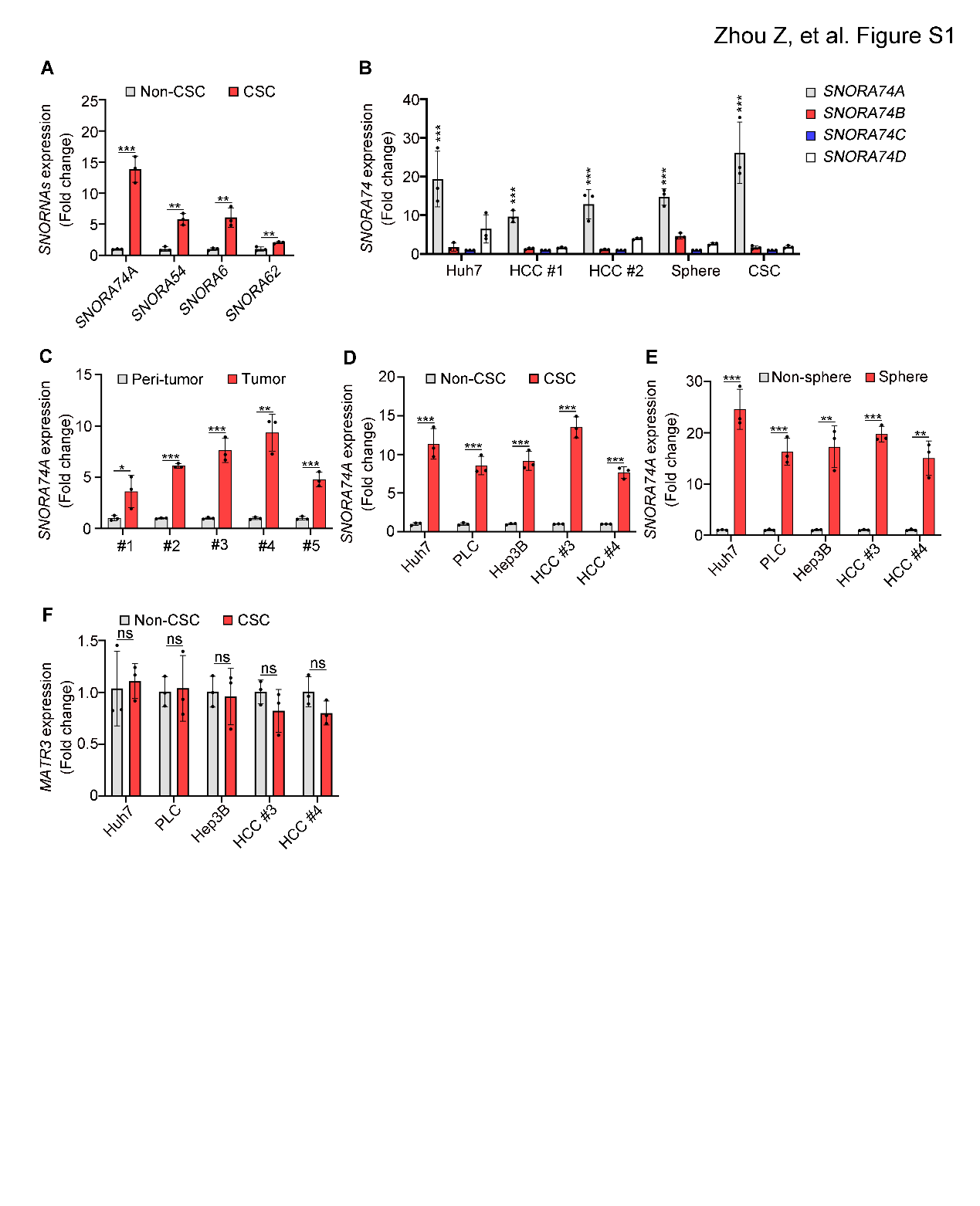


**Figure S1. *SNORA74A* is highly upregulated in human HCC tumor tissues and liver CSCs.**

(A) Four upregulated H/ACA box snoRNAs were detected in liver CSCs and non-CSCs by qRT-PCR. Data are presented as means ± SD. n = 3 for each group. Unless otherwise specified in this study, qRT-PCR data were normalized to endogenous 18S rRNA. (B) qRT-PCR analysis was conducted to assess expression of *SNORA74A* and its homologous transcripts in Huh7 cells, HCC samples, spheres, and liver CSCs. Data are presented as means ± SD. n = 3 for each group. (C-E) Expression levels of *SNORA74A* in HCC tumors and peri-tumors (C), in CD13^+^CD133^+^ CSCs and CD13^-^CD133^-^ non-CSCs (D), and in oncospheres and non-spheres (E). Results are presented as means ± SD. n = 3 for each group. (F) Expression levels of *MATR3* in liver CSCs and non-CSCs. Data are shown as means ± SD. n = 3 for each group. ** *P* < 0.01; *** *P* < 0.001; not significant (ns) *P* > 0.05 by two-tailed Student’s T test. Data are representative of at least three independent experiments.


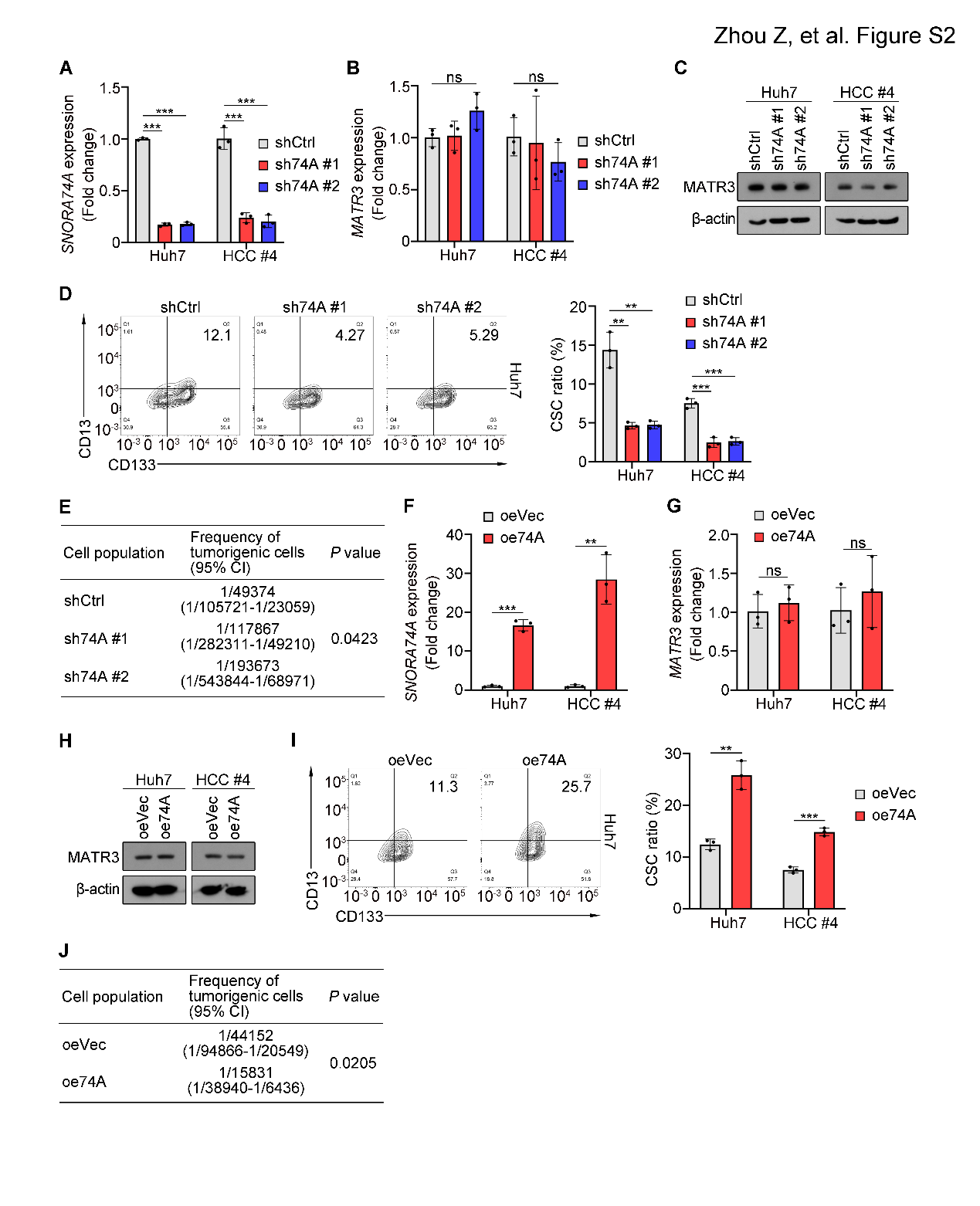


**Figure S2. Generation of *SNORA74A* depleted and overexpressed HCC cells.**

(A) qRT-PCR analysis evaluated depletion effect of *SNORA74A* in Huh7 cells and human primary HCC cells. Data are shown as means ± SD. n = 3 for each group. (B, C) Expression levels of *MATR3* in *SNORA74A* depleted Huh7 cells and human primary HCC cells were examined by qRT-PCR (B) and Western blot (C). Data are shown as means ± SD. n = 3 for each group. (D) FACS analysis assessed liver CSCs (CD13^+^CD133^+^) in *SNORA74A* depleted and control cells. Data are shown as means ± SD. n = 3 for each group. (E) Tumorigenic cell frequencies in *SNORA74A* depleted and control cells were analyzed by a limiting dilution assay (http://bioinf.wehi.edu.au/software/elda/). Data are shown as means and 95% confidence intervals (n = 8). (F) qRT-PCR analysis evaluated overexpression effect of *SNORA74A* in Huh7 cells and human primary HCC cells. Data are shown as means ± SD. n = 3 for each group. (G, H) Expression levels of *MATR3* in *SNORA74A* overexpressed Huh7 cells and human primary HCC cells were examined by qRT-PCR (G) and Western blot (H). Data are shown as means ± SD. n = 3 for each group. (I) FACS analysis assessed liver CSCs (CD13^+^CD133^+^) in *SNORA74A* overexpression and control cells. Data are shown as means ± SD. n = 3 for each group. (J) Tumorigenic cell frequencies in *SNORA74A* overexpressed and control cells were analyzed by a limiting dilution assay. Data are shown as means and 95% confidence intervals (n = 8). ** *P* < 0.01; *** *P* < 0.001; not significant (ns) *P* > 0.05 by two-tailed Student’s T test. Data are representative of at least three independent experiments.


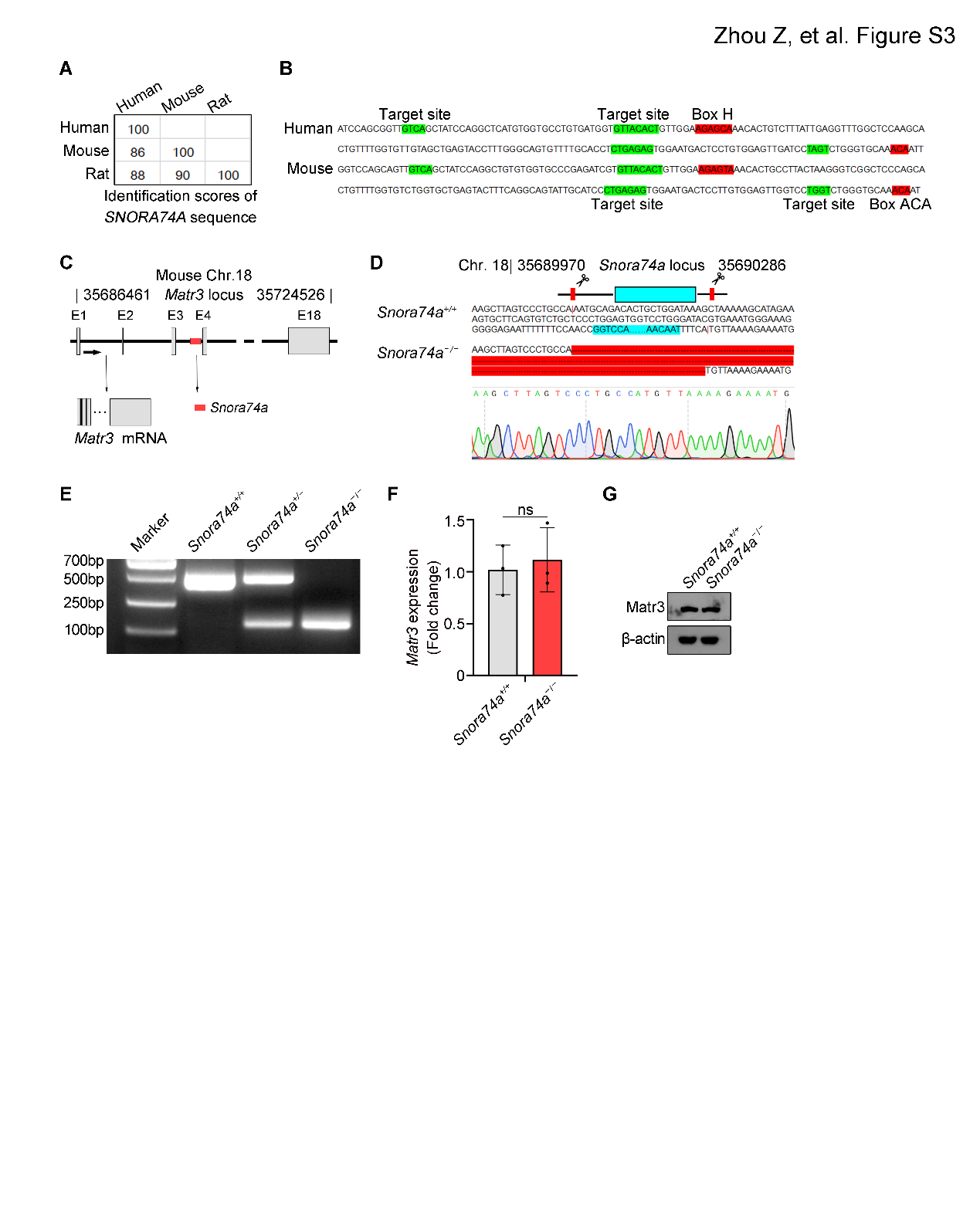


**Figure S3. Generation of *Snora74a* knockout mice.**

(A) Homology analysis of *SNORA74A* across human, mouse, and rat genomes. (B) Sequences, target sites, and H/ACA box of *SNORA74A* in humans and mice. (C) Schematic representation of mouse *Snora74a*. Arrow indicates the position of *Snora74a* in the linear gene locus. *Snora74a* marks with a red square. E1, exon #1. (D) Schematic representation for *Snora74a* KO mice (upper panel). The gene locus of *Snora74a* was deleted using CRISPR/Cas9 system. *Snora74a* KO mice were verified by DNA sequencing (lower panel). (E) Gel electrophoresis validation of *Snora74a* KO mice. (F, G) Expression levels of *Matr3* in *Snora74a* KO mice were examined by qRT-PCR (F) and Western blot (G). Data are shown as means ± SD. n = 3 for each group. Not significant (ns) *P* > 0.05 by two-tailed Student’s T test. Data are representative of at least three independent experiments.


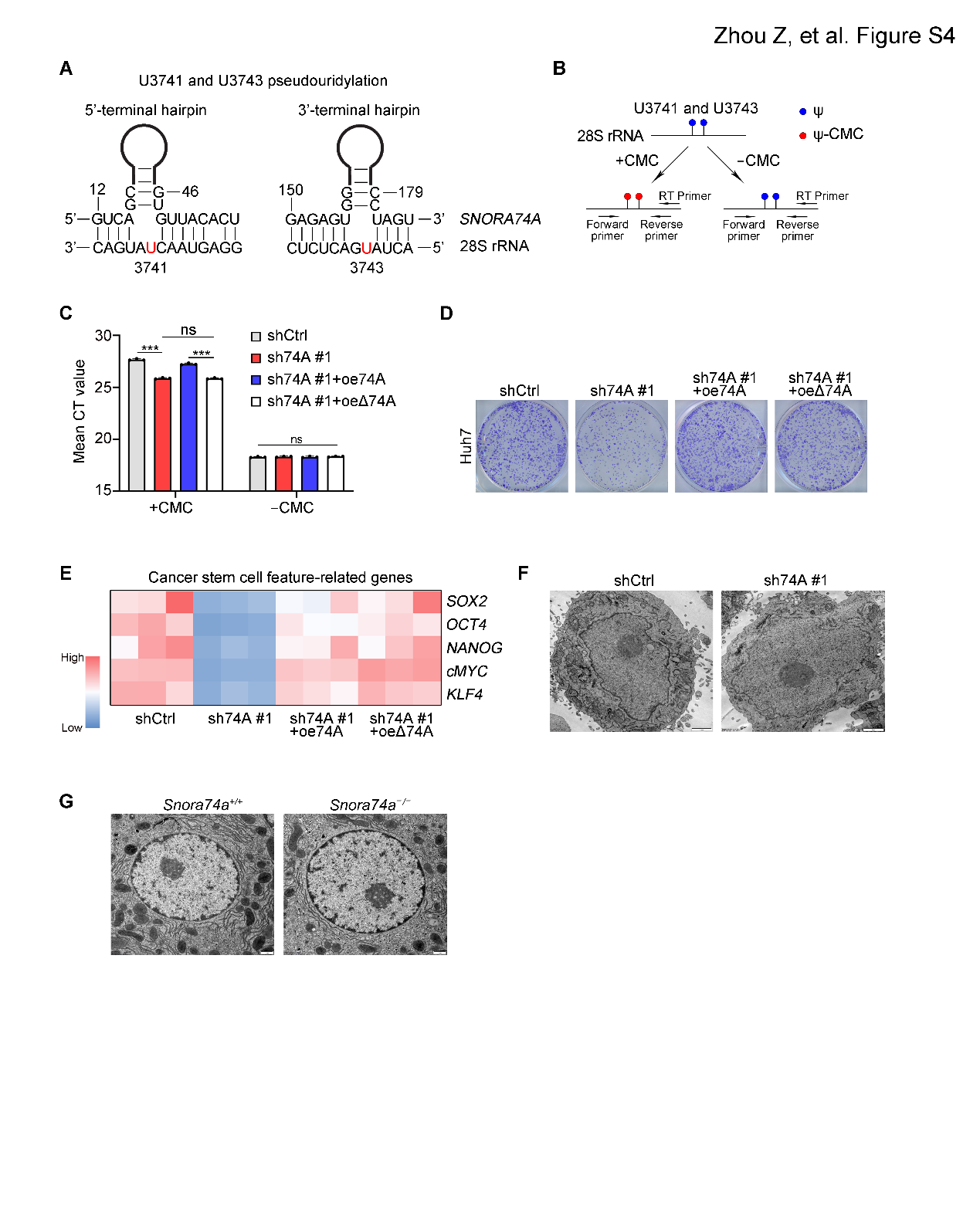


**Figure S4.** ***SNORA74A* drives self-renewal of liver CSCs in a canonical function independent manner.**

(A) Diagram of *SNORA74A* binding regions on 28S rRNA. (B) Diagram of pseudouridine modification sites analysis using qRT-PCR and CMC. (C) Mean CT values with or without CMC in *SNORA74A* depletion, *SNORA74A* rescued, and *SNORA74A* binding region deletion (oe△74A) rescued conditions. Data are shown as means ± SD. n = 3 for each group. (D) Representative images of clone formation capability in *SNORA74A* depletion, *SNORA74A* rescued, and *SNORA74A* binding region deletion (oe△74A) rescued conditions. (E) Expression profiles of CSC-related genes in control, *SNORA74A* depletion, *SNORA74A* rescued, or oe△74A. n = 3 for each group. (F) Representative TEM images of ultrastructure in *SNORA74A* depleted and control HCC cells. Scale bar, 2 μm. (G) Representative TEM images of ultrastructure in hepatocytes from *Snora74a^+/+^* and *Snora74a^-/-^* mice. Scale bar, 1 μm. *** *P* < 0.001; not significant (ns) *P* > 0.05 by two-tailed Student’s t test. Data are representative of at least three independent experiments.


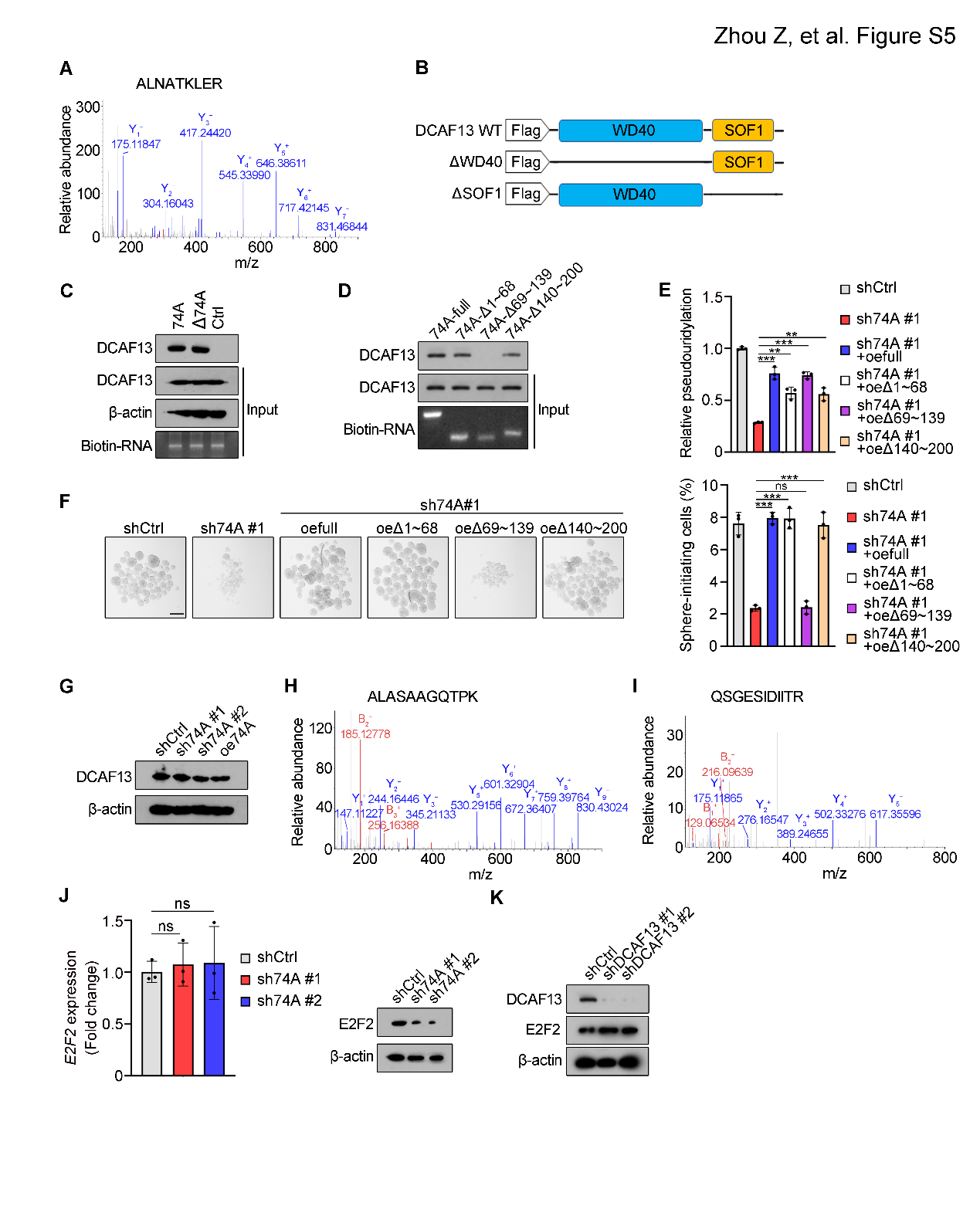


**Figure S5.** ***SNORA74A* regulates self-renewal of liver CSCs by interacting with DCAF13.**

(A) Representative MS profile of DCAF13 with corresponding peptide sequences listed at the top of chart. (B) Schematic diagram of DCAF13 protein domains. WD40 domain (59~353 aa); SOF1 domain (354~441 aa). △WD40, lacking 59~353 aa of DCAF13; △SOF1, lacking 354~441 aa of DCAF13. (C) RNA-pulldown enrichment followed by Western blotting using linear biotin-labeled *SNORA74A* or *SNORA74A* binding region deletion (oe△74A) in liver CSCs lysates. (D) *SNORA74A* truncated fragments were incubated with cell lysates, followed by RNA pulldown assay and Western blot. (E) Relative pseudouridine modification levels at U3741 and U3743 of 28S rRNA in control, *SNORA74A* depletion, and *SNORA74A* truncated fragments rescued conditions. Data are shown as means ± SD. n = 3 for each group. (F) Oncosphere formation assays in control, *SNORA74A* depletion, and *SNORA74A* truncated fragments rescued (left panel). Scale bar, 100 μm. Oncosphere formation rates were assessed (right panel). Data are presented as means ± SD. n = 3 for each group. (G) Protein levels of DCAF13 in control, *SNORA74A* depletion, and *SNORA74A* overexpressed liver CSCs were assessed via Western blotting. (H, I) Representative MS profiles of E2F2 (H) and DDB1 (I) with corresponding peptide sequences listed at the top of chart. (J) Expression levels of *E2F2* in *SNORA74A* depleted liver CSCs examined by qRT-PCR (left panel) and Western blot (right panel). Data are shown as means ± SD. n = 3 for each group. (K) Expression levels of DACF13 and E2F2 in DCAF13 depleted liver CSCs by Western blotting. * *P* < 0.05; ** *P* < 0.01; *** *P* < 0.001; not significant (ns) *P* > 0.05 by two-tailed Student’s T test. Data are representative of at least three independent experiments.


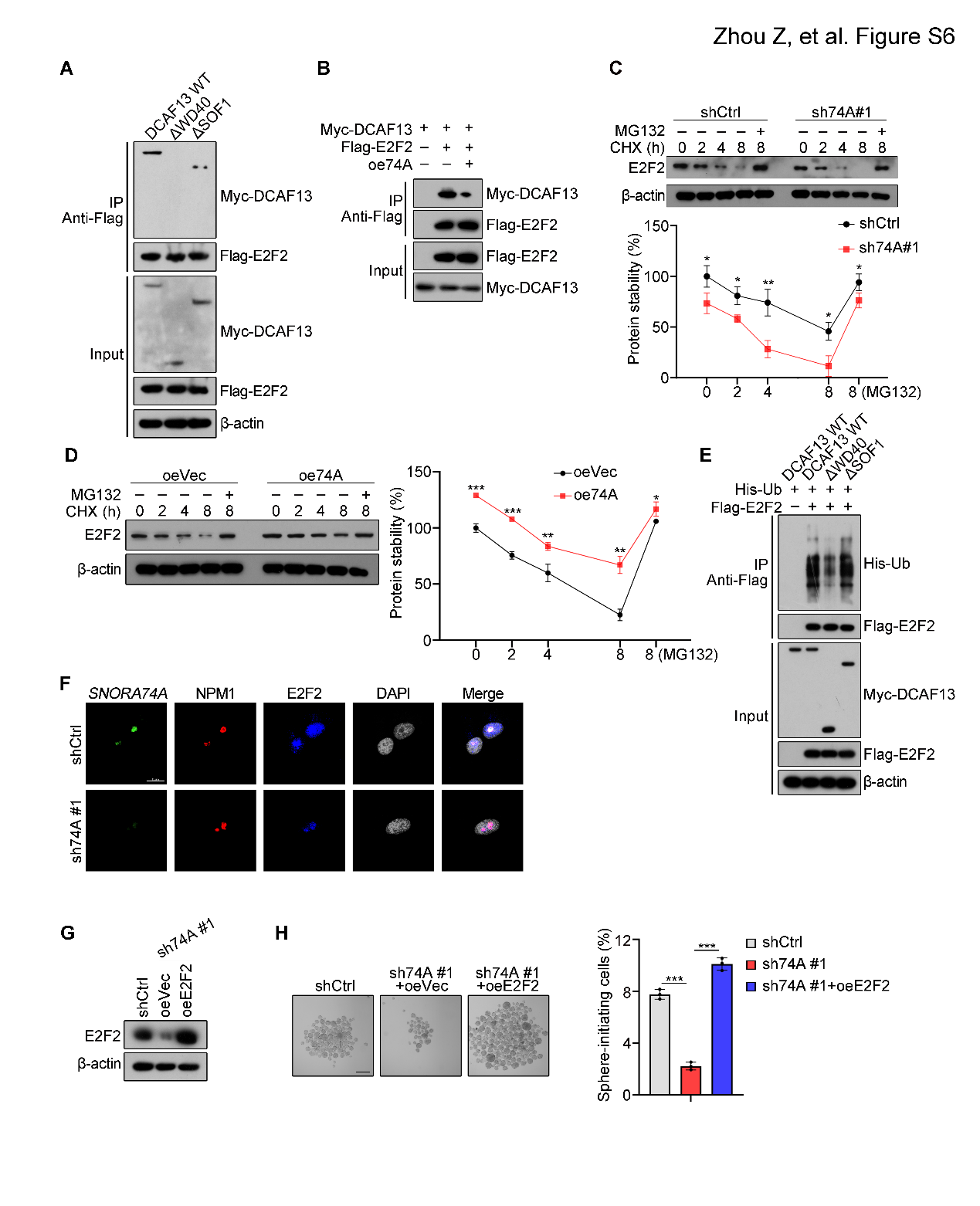


**Figure S6.** ***SNORA74A* reduces the ubiquitination level of E2F2 by inhibiting the interaction between E2F2 and DCAF13.**

(A) Domain mapping of DCAF13 protein with Flag-tagged E2F2, followed by Co-IP experiment and Western blotting with anti-Flag or anti-Myc antibodies. (B) Myc-tagged DCAF13 and Flag-tagged E2F2 were co-transfected into control or *SNORA74A* overexpressed liver CSCs for 48 h. Cell lysates were immunoprecipitated with anti-Flag antibody, followed by Western blotting with anti-Flag or anti-Myc antibodies. (C) 1 × 10^6^ *SNORA74A* depleted or control Huh7 cells were treated with CHX (20 μg/ml) and MG132 (10 μM). At different time points, equal amounts of cells were sampled and protein levels were analyzed by Western blotting (upper panel). Percentages of remaining protein amounts were normalized to control cells and calculated as means ± SD (n = 3) (lower panel). (D) 1 × 10^6^ *SNORA74A* overexpressed or control Huh7 cells were treated with CHX (20 µg/ml) and MG132 (10 µM). At different time points, equal amounts of cells were sampled and protein levels were analyzed by Western blotting with anti-E2F2 antibody (left panel). Percentages of remaining protein amounts were normalized to control cell amounts and calculated as means ± SD (n = 3) (right panel). (E) Myc-tagged DCAF13 domains, Flag-tagged E2F2, and His-tagged ubiquitin were co-transfected into liver CSCs for 48 h. Cell lysates were incubated with anti-Flag antibody for immunoprecipitation, followed by Western blotting. (F) Representative immunofluorescence staining of *SNORA74A*, E2F2 and NPM1 in control and *SNORA74A* depleted liver CSCs. *SNORA74A* was visualized by RNA FISH. Scale bar, 15 μm. (G) Overexpression of E2F2 in *SNORA74A* depleted cells was assayed by immunoblotting. (H) E2F2 overexpression restored oncosphere formation capability reduced by *SNORA74A* depletion (left panel). Scale bar, 200 μm. Oncosphere formation rates were assessed (right panel). Data are presented as mean ± SD. n = 3 for each group. * *P* < 0.05; ** *P* < 0.01; *** *P* < 0.001 by two-tailed Student’s t test. Data are representative of at least three independent experiments.

**
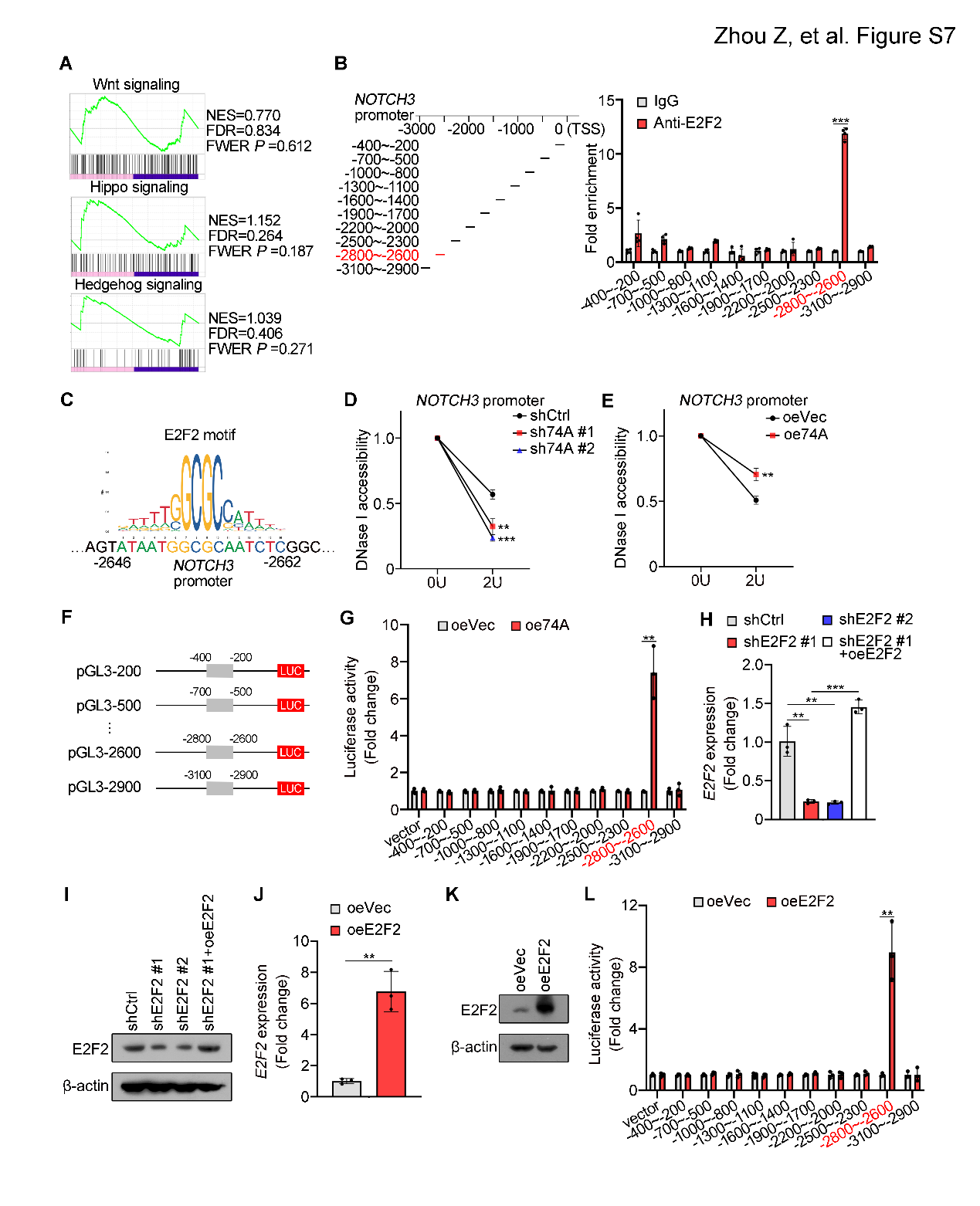
**

**Figure S7.** **E2F2 binds to the promoter region of *NOTCH3* gene.**

(A) Differentially expressed genes were not enriched in Wnt, Hippo, and Hedgehog signaling pathways. NES, normalized enrichment score; FDR, false discovery rate; FWER, familywise error rate. (B) ChIP assays were conducted to identify binding regions of E2F2 on the *NOTCH3* promoter in liver CSCs, followed by qPCR analysis, with IgG enrichment used as a control. Results are presented as means ± SD. n = 3 for each group. (C) E2F2-binding motif was predicted in *NOTCH3* promoter using JASPAR. (D) DNase I assay of chromatin accessibility in *NOTCH3* promoters of *SNORA74A* depleted or control liver CSCs by qRT-PCR. n = 3 for each group. Results are shown as means ± SD. (E) DNase I assay of chromatin accessibility in *NOTCH3* promoters of *SNORA74A* overexpressed or control liver CSCs by qRT-PCR. n = 3 for each group. Results are shown as means ± SD. (F) Diagram shows strategy of dividing the *NOTCH3* promoter into 10 regions for construction into pGL3 vector. (G) Luciferase reporter assay was performed in *SNORA74A* overexpressed or control liver CSCs. Results are shown as means ± SD. n = 3 for each group. (H, I) Expression levels of *E2F2* were assessed in control, *E2F2* depleted or *E2F2* rescued liver CSCs using qRT-PCR (H) and Western blotting (I). Results are shown as means ± SD. n = 3 for each group. (J, K) Expression levels of *E2F2* were assessed in control and *E2F2* overexpressed liver CSCs using qRT-PCR (J) and Western blotting (K). Results are shown as means ± SD. n = 3 for each group. (L) Luciferase reporter assay was performed in *E2F2* overexpressed or control liver CSCs. Results are shown as means ± SD. n = 3 for each group. * *P* < 0.05; ** *P* < 0.01; *** *P* < 0.001 by two-tailed Student’s t test. Data are representative of at least three independent experiments.


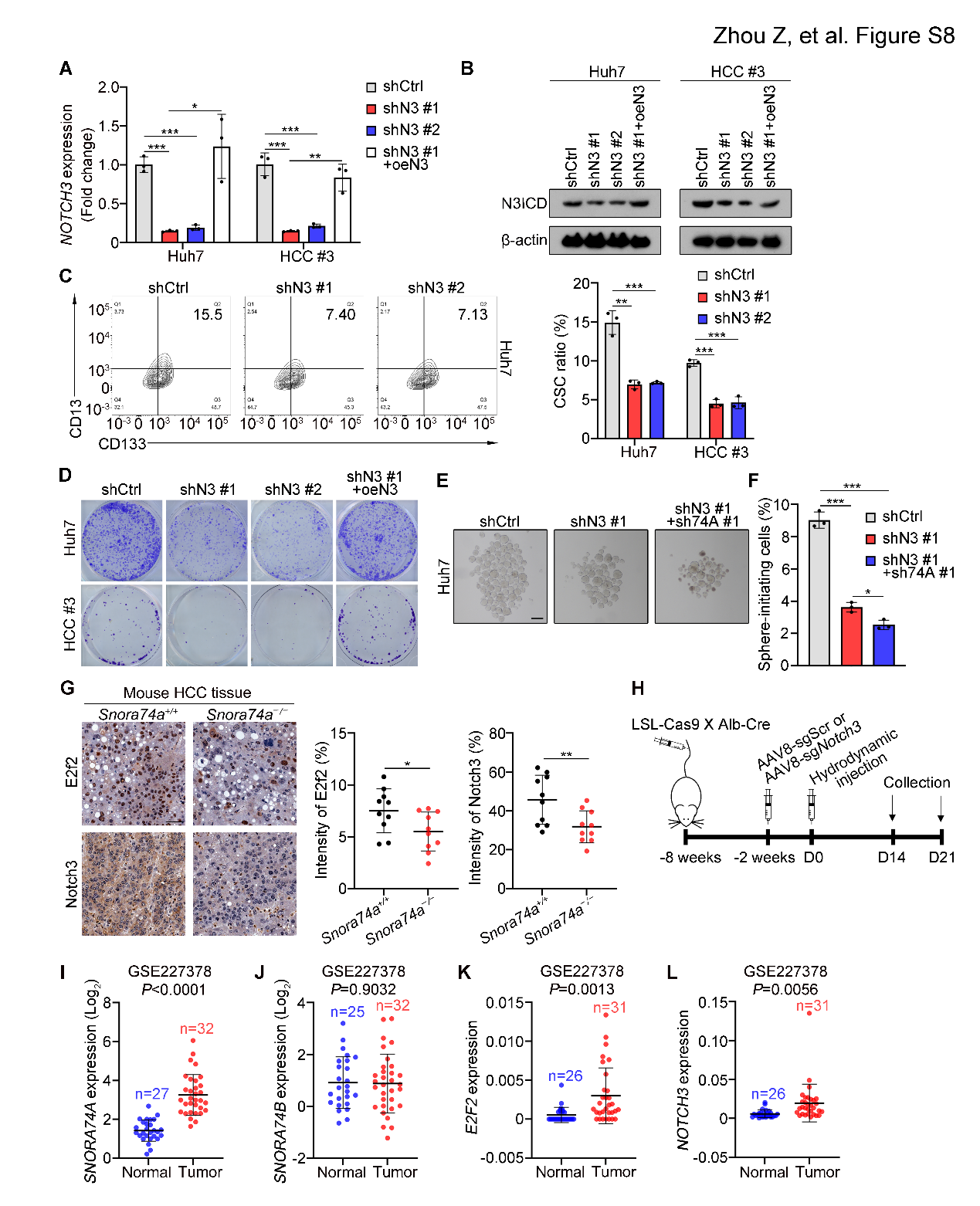


**Figure S8.** ***NOTCH3* promotes self-renewal of liver CSCs.**

(A, B) Expression levels of *NOTCH3* were assessed in control, *NOTCH3* depleted and *NOTCH3* rescued Huh7 cells and human primary HCC cells using qRT-PCR (A) and Western blotting (B). Results are shown as means ± SD. n = 3 for each group. (C) FACS analysis assessed liver CSCs (CD13+CD133+) in *NOTCH3* depleted and control cells. Data in right panel are shown as means ± SD. n = 3 for each group. (D) Representative images of clone formation capability in *NOTCH3* depleted or control Huh7 cells and human primary HCC cells are shown. (E) Oncosphere formation assays in control, *NOTCH3* depletion, or *NOTCH3* and *SNORA74A* depletion HCC cells. Representative images were shown. Scale bar, 100μm. (F) Oncosphere formation rates were assessed. Data are presented as means ± SD. n = 3 for each group. (G) Immunohistochemical staining of E2f2 and Notch3 were performed in HCC samples of *Snora74a* KO or WT mice (left panel). Scale bar, 50 μm. Protein expression intensities were assessed (right panel). 10 visual fields were counted using ImageJ. Data are presented as mean ± SD. (H) Schematic diagram illustrates induction of liver tumors in control or sg*Notch3* mice. (I-L) Expression levels of *SNORA74A* (I), *SNORA74B* (J), *E2F2* (K), and *NOTCH3* (L) in HCC samples provided by GSE227378. Data are presented as means ± SD. * *P* < 0.05; ** *P* < 0.01; *** *P* < 0.001 by two-tailed Student’s t test. Data are representative of at least three independent experiments.

**
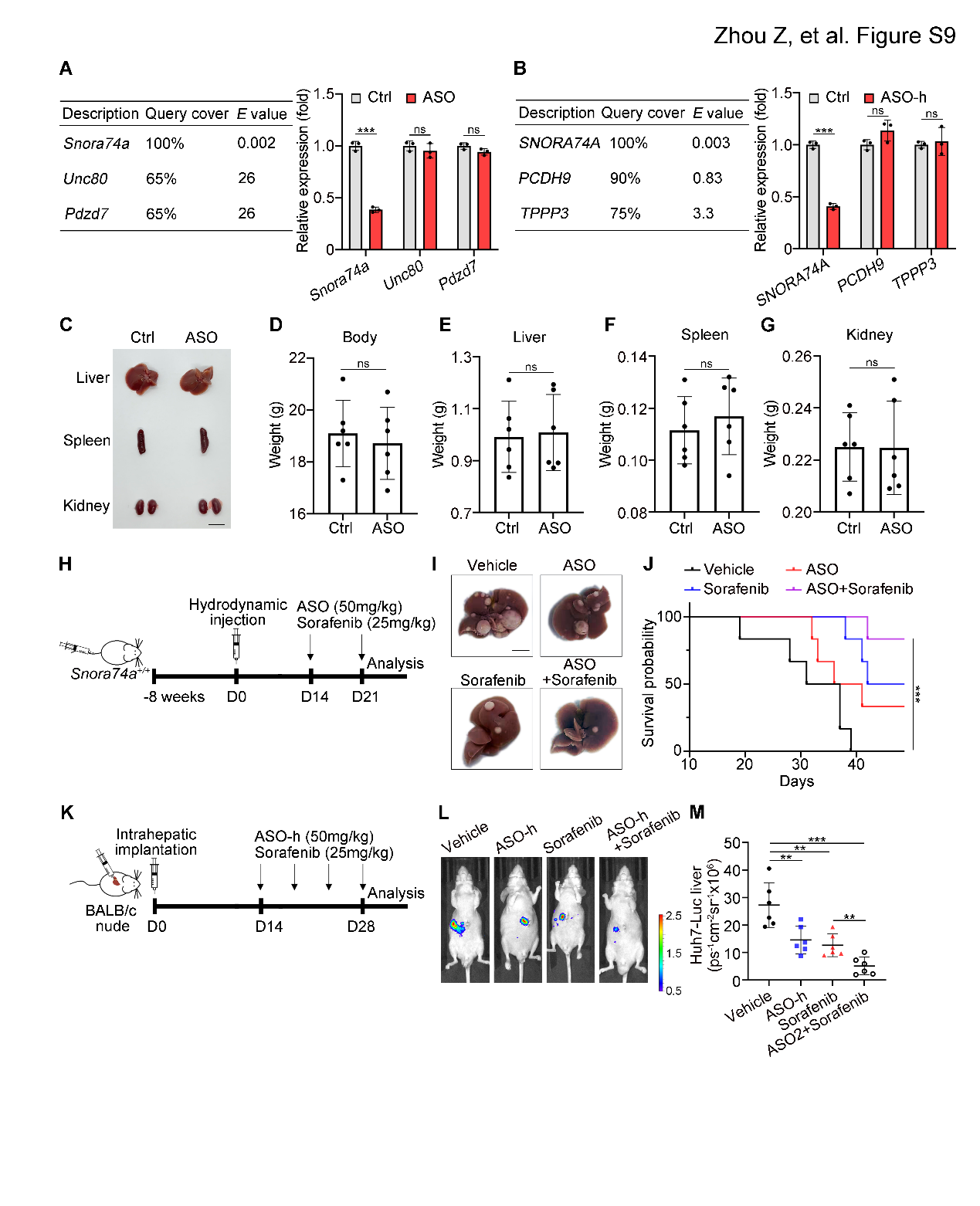
**

**Figure S9.** **DAPT with ASOs against *SNORA74A* shows a synergistic anti-tumor effect.**

(A) ASO sequence was aligned using BLAST (left panel), and expression levels of target genes in tumors after delivery were detected using qRT-PCR (right panel). n = 3 for each group. Data are presented as means ± SD. (B) ASO-h sequence was aligned using BLAST (left panel), and detected using qRT-PCR (right panel). n = 3 for each group. Data are presented as means ± SD. (C) Representative images of organs from WT mice after ASO delivery. Scale bar, 1 cm. (D-G) No significant change in body (D), liver (E), spleen (F) or kidney (G) weights after treatment with ASOs. n = 6 for each group. Data are presented as means ± SD. (H) Schematic representation of ASO plus sorafenib treatment timeline in mice. (I, J) Effects with treatment of ASOs combined with sorafenib in tumor-bearing mice after hydrodynamic injection. Representative liver images (I) and survival analysis (J) are shown. n = 6 for each group. Scale bars, 1 cm. (K) Schematic representation of ASO-h plus sorafenib therapy method. (L, M) Orthotopic human liver tumor growth was imaged via luciferase signals. Representative images are shown (L), and statistical results are shown as means ± SD (M). n = 6 for each group. * *P* < 0.05; ** *P* < 0.01; *** *P* < 0.001; not significant (ns) *P* > 0.05 by two-tailed Student’s T test. Data are representative of at least three independent experiments.


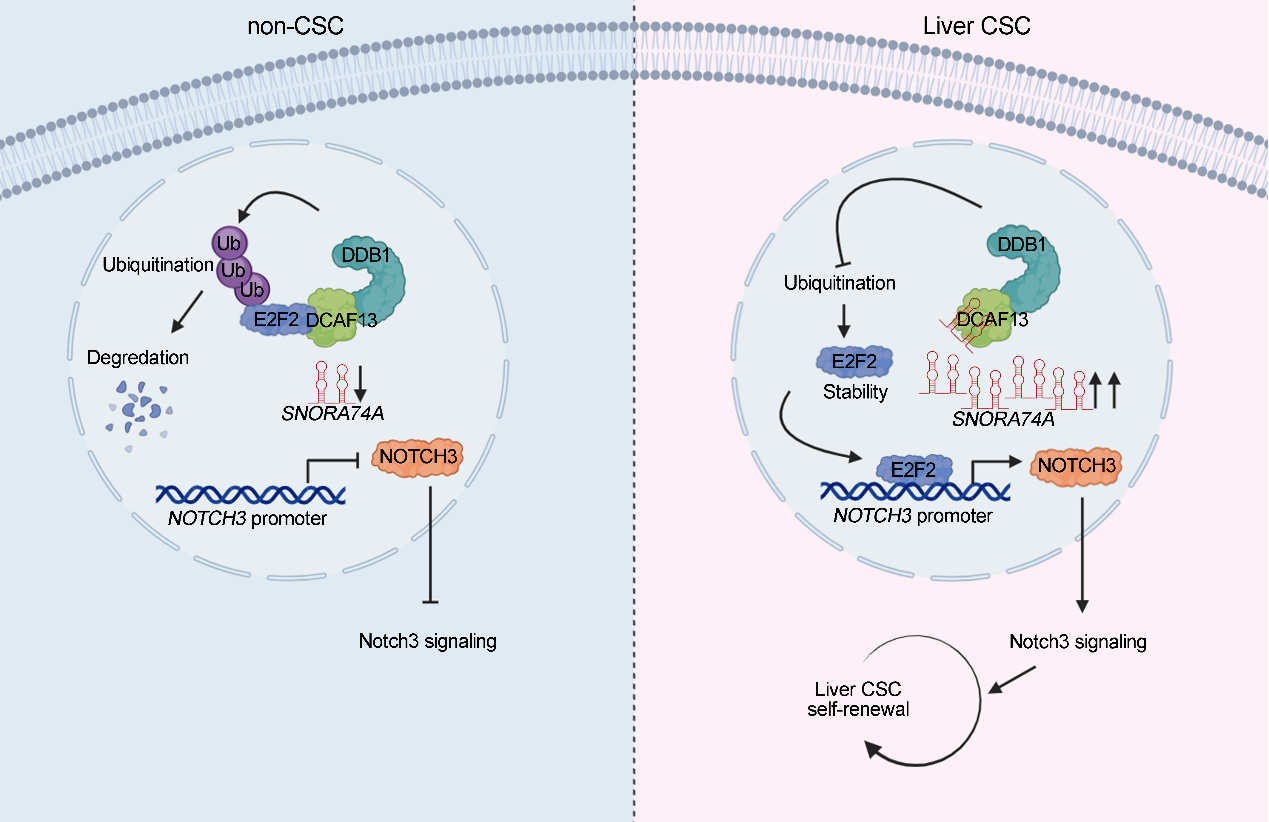


**Figure S10. Graphic abstract of this study.**

Highly expressed *SNORA74A* in liver CSCs binds DCAF13 to prevent formation of the DCAF13-contained E3 ligase for K48-linked ubiquitination of E2F2. Stable E2F2 induces *NOTCH3* transcription to initiate Notch3 signaling activation, leading to self-renewal of liver CSCs and hepatocarcinogenesis. In non-CSCs, lowly expressed *SNORA74A* causes recruitment of the DCAF13-contained E3 ligase for E2F2 ubiquitination, resulting in E2F2 degradation.

**Table S1. Materials used in this study**

| Reagents or Resource | Source | Identifier |
| --- | --- | --- |
| Antibodies | | |
| Histone H3 | CST | Cat: 4499 |
| β-actin | Sigma-Aldrich | Cat: A1978 |
| EEA1 | Santa Cruz | Cat: SC-6415 |
| Nucleophosmin  Nucleolin | Abcam  Proteintech | Cat: ab52644  Cat: 10556-1-AP |
| Lamin A/C | MCE | Cat: HY-P80204 |
| MATR3 | Solarbio | Cat: K007098P |
| DCAF13 | Abcam | Cat: ab195121 |
| E2F2 | Sangon Biotech | Cat: D120572 |
| Ubiquitin | Proteintech | Cat: 10201-2-AP |
| K48-linkage Ubiquitin | ABclonal | Cat: A3606 |
| Flag-tag  His-tag | Ray Antibody  Ray Antibody | Cat: RM1002  Cat: RM1001 |
| HA-tag | Proteintech | Cat: 51064-2-AP |
| c-Myc-tag | Sungene Biotech | Cat: KM8003 |
| PCNA | Proteintech | Cat: 10205-2-AP |
| NOTCH3 | Abcam | Cat: ab252845 |
| NOTCH3 | Abcam | Cat: ab300527 |
| NOTCH3 | Solarbio | Cat: K003596P |
| HEY1  HEY2  HES5 | Proteintech  Biodragon  Solarbio | Cat: 19929-1-AP  Cat: BD-PT2230  Cat: K009612P |
| 594 Donkey anti-Rabbit, Secondary Antibody | Invitrogen | Cat: A21207 |
| 647 Donkey anti-Mouse, Secondary Antibody | Invitrogen | Cat: A31571 |
| 488 Donkey anti-Rabbit, Secondary Antibody | Invitrogen | Cat: A21206 |
| FITC-conjugated CD13 | eBioscience | Cat: 11-0138 |
| PE-conjugated CD133 | Miltenyi Biotec | Cat: 130-098-826 |
| Alexa Fluor® 555 Donkey anti-rabbit IgG | Biolegend | Cat: 406412-100ug |
| Alexa Fluor594 anti-Nucleophosmin | Biolegend | Cat: **686803** |
| HRP Streptavidin | Biolegend | Cat: 405210 |
| Chemicals, Peptides, and Recombinant Proteins | | |
| T7 RNA polymerase | Roche | Cat: 10881767001 |
| Biotin RNA Labeling Mix | Roche | Cat: 11685597910 |
| Blocking Reagent | Roche | Cat: 11096176001 |
| N2 supplement | Sigma-Aldrich | Cat: 17502-048 |
| Streptavidin, immobilized on Agarose CL-4B | Sigma-Aldrich | Cat: **85881** |
| EGF | Sigma-Aldrich | Cat: E5036-200UG |
| DAPI | Sigma-Aldrich | Cat: 28718-90-3 |
| DNase I | NEB | Cat: M0303S |
| M5 RNase inhibitor | Mei5 Biotech | Cat: MF010-05 |
| Protein A/G Magnetic Beads | MCE | Cat: HY-K0202 |
| Collagenase, Type 4 | Worthington | Cat: **LS004188** |
| bFGF | Millipore | Cat: GF446-50UG |
| B27 | InvitroGen | Cat: 17504-044 |
| Puromycin | InvivoGen | Cat: ant-pr-1 |
| Plasmocin™ prophylactic | InvivoGen | Cat: ant-mpp |
| DEPC | aladdin | Cat: **D105557-100g** |
| Corning Matrigel Basement Membrane Matrix | BioCoat | Cat: **354234** |
| LIPOFECTAMINE 3000 | Thermo | Cat: **L3000015** |
| T4 Polynucleotide Kinase | TaKaRa | Cat: **2021B** |
| Cycloheximide  MG132  D-Luciferin sodium salt  N-Cyclohexyl-N′-(2-morpholinoethyl)carbodiimide methyl-p-toluenesulfonate | MCE  Cayman  Aladdin  Sigma-Aldrich | **Cat: HY-12320**  **Cat: 10012628**  Cat: D115509  **Cat: C106402** |
| BICINE  Sorafenib  DAPT | Beyotime  MCE  MCE | **Cat: ST2227**  **Cat: HY-10201**  **Cat: HY-13027** |
| Critical Commercial Assays | | |
| Fluorescein TSA Fluorescence System Kit | APExBIO | Cat: K1050-100 |
| SYBR Green reaction system  NovoRec^®^ plus One step PCR Cloning Kit | TIANGEN  Novoprotein | Cat: FP205  Cat: NR005-01A |
| Chemiluminescent Nucleic Acid Detection Module | Thermo | Cat: 89880 |
| NE-PER Nuclear and cytoplasmic extraction | Thermo | Cat: 78833 |
| Supersignal West pico plus  Chemiluminescent EMSA Kit | Thermo  Beyotime | Cat: 34577  Cat: GS009 |
| Rabbit two-step test kit | ZSGB-BIO | Cat: PV-9001-18ml |
| Universal Virus Concentration Kit | Beyotime | Cat: C2901S |
| Ultra-low attachment plates | Corning | Cat: 3471 |
| Dual Luciferase Reporter Gene Assay Kit | Beyotime | Cat: RG027 |
| Experimental Models: Cell Lines | | |
| Huh7 | provided by Dr. Zeguang Han  (Shanghai Jiaotong University School of Medicine, China) | |
| PLC/PRF/5 |  |  |
| Hep3B |  |  |

**Table S2. shRNA sequences used in this study**

| Genes | shRNA sequences |
| --- | --- |
| *SNORA74A-1#* | 5’- GTCAGCTATCCAGGCTCATGT-3' |
| *SNORA74A-2#* | 5’- GCCTGTGATGGTGTTACACTG-3' |
| *SNORA54-1#* | 5’- GAGCATGTTCTGTAACCCGTT-3' |
| *SNORA54-2#* | 5’- CTGGTGTGATGCTATGGGTT-3' |
| *SNORA6-1#* | 5’- GCTAACACCAGTAGAGCTTGC-3' |
| *SNORA6-2#* | 5’- GCCTCTATGACTGGAGTTTGG-3' |
| *SNORA62-1#* | 5’- GAGCTTGGAGTTGAGGCTA-3’ |
| *SNORA62-2#* | 5’- GGCCGATGAACTCGCAAGT-3' |
| *SNORD88B-1#* | 5’- GGGACCCCGTGATGTCCAG-3’ |
| *SNORD88B-2#*  *DCAF13-1#*  *DCAF13-2#* | 5’- TGACTGCCCCTGAGGACAC-3'  5’- GGACAATTATGTCCGCGAAAC-3'  5’- GGAGTCAATTGCTTGGCAAAG-3' |
| *E2F2-1#* | 5’- GCTCACCAAGAAGTTCATTTA-3' |
| *E2F2-2#* | 5’- GCATCTATGACATCACCAACG-3' |
| *NOTCH3-1#* | 5’- GCATGAAGAACATGGCCAAGG-3' |
| *NOTCH3-2#* | 5’- GGAGCCAATGCCAACTGAAGA-3' |

**Table S3.** **sgRNAs for mouse knockout construction and primers for genotyping**

| Genes | sgRNA sequences | |
| --- | --- | --- |
| *Snora74a* (up) | 5’-CTGCCAAATGCAGACACTGCTGG-3' | |
| *Snora74a* (down) | 5’-ATGTTAAAAGAAAATGGGACTGG-3' | |
| Primers | **Sequences** | |
| *Snora74a* identification (Forward) | | 5’-TTTTGCCATTCCAACCGAGG-3' |
| *Snora74a* identification (Reverse) | 5’-TGCATAGCCAAGTCCCAGTC-3' | |

**Table S4. qPCR primers used in this study**

| Genes | Primer sequences |
| --- | --- |
| *18S (Forward)* | 5’-AACCCGTTGAACCCCATT-3’ |
| *18S (Reverse)* | 5’-CCATCCAATCGGTAGTAGCG-3’ |
| *ACTB (Forward)* | 5’-GTCACCAACTGGGACGACAT-3’ |
| *ACTB (Reverse)* | 5’-AGGGATAGCACAGCCTGGAT-3’ |
| *U1 (Forward)* | 5’-ATACTTACCTGGCGCAGGGG-3’ |
| *U1 (Reverse)* | 5’-AGGAGATAGCGCGAACGCA-3’ |
| *GAPDH (Forward)* | 5’-ACAACTTTGGTATCGTGGAAGG-3’ |
| *GAPDH (Reverse)* | 5’-GCCATCACGCCACAGTTTC-3’ |
| *HMBS (Forward)* | 5’-AGCTTGCTCGCATACAGACG-3’ |
| *HMBS (Reverse)* | 5’-AGCTCCTTGGTAAACAGGCTT-3’ |
| *MATR3 (Forward)* | 5’-ATCAATGGAGCAAGTCACAGTC-3’ |
| *MATR3 (Reverse)* | 5’-TGCAACATGAATGGATCACCC-3’ |
| *Matr3 (Forward)* | 5’-GTCATCTCTCGGTAGGGATTCA-3’ |
| *Matr3 (Reverse)* | 5’-TGCTGGCATACTTAAAGACTGG-3’ |
| *E2F2 (Forward)* | 5’-CGTCCCTGAGTTCCCAACC-3’ |
| *E2F2 (Reverse)* | 5’-GCGAAGTGTCATACCGAGTCTT-3’ |
| *NOTCH1 (Forward)* | 5’-TTGGGAGGAGCAGATTTTTG-3’ |
| *NOTCH1 (Reverse)* | 5’-CACTGGCATGACACACAACA-3’ |
| *NOTCH2 (Forward)* | 5’-CAACTCGATGAGTGTGCGTC-3’ |
| *NOTCH2 (Reverse)* | 5’-ATGCCCTGGATGGAAAATGGA-3’ |
| *NOTCH3 (Forward)* | 5’-AGATTCTCATCCGAAACCGCTCTA-3’ |
| *NOTCH3 (Reverse)* | 5’-GGGGTCTCCTCCTTGCTATCCTG-3’ |
| *NOTCH4 (Forward)* | 5’-GCGGAGGCAGGGTCTCAACGGATG-3’ |
| *NOTCH4 (Reverse)* | 5’-AGGAGGCGGGATCGGAATGT-3’ |
| *HES1 (Forward)* | 5’-TCAACACGACACCGGATAAAC-3’ |
| *HES1 (Reverse)* | 5’-GCCGCGAGCTATCTTTCTTCA-3’ |
| *HES5 (Forward)* | 5’-AGAGAAAAACCGACTGCGGA-3’ |
| *HES5 (Reverse)* | 5’-GACGAAGGCTTTGCTGTGC-3’ |
| *HES6 (Forward)* | 5’-AGCAGGAGCCTGACTCAGTT-3’ |
| *HES6 (Reverse)*  *HEY1 (Forward)*  *HEY1 (Reverse)*  *HEY2 (Forward)*  *HEY2 (Reverse)*  *NRARP (Forward)*  *NRARP (Reverse)*  *MYC (Forward)*  *MYC (Reverse)*  *Unc80 (Forward)*  *Unc80 (Reverse)*  *Pdzd7 (Forward)*  *Pdzd7 (Reverse)*  *PCDH9 (Forward)*  *PCDH9 (Reverse)*  *TPPP3 (Forward)*  *TPPP3 (Reverse)*  *SOX2 (Forward)*  *SOX2 (Reverse)*  *OCT4 (Forward)*  *OCT4 (Reverse)*  *NANOG (Forward)*  *NANOG (Reverse)*  *cMYC (Forward)*  *cMYC (Reverse)*  *KLF4 (Forward)*  *KLF4 (Reverse)* | 5’-AGCTCCTGAACCATCTGCTC-3’  5’-GTTCGGCTCTAGGTTCCATGT-3’  5’-CGTCGGCGCTTCTCAATTATTC-3’  5’-AAGGCGTCGGGATCGGATAA-3’  5’-AGAGCGTGTGCGTCAAAGTAG-3’  5’-CACGGGGTGATCACTGCTAA-3’  5’-CGCTGGGCTACAGGTCAATA-3’  5’-GGCTCCTGGCAAAAGGTCA-3’  5’-CTGCGTAGTTGTGCTGATGT-3’  5’-TCAGTTTGGAGGTACAGACCG-3’  5’-CCAGTCGGAAGGTGAGATCA-3’  5’-TCTCTGGGGGCATCGAGTC-3’  5’-GCTCAAAGCCAGCCTGTAGAT-3’  5’-CTGCTCTGATTGCCTGTTTAAGG-3’  5’-ACCAGTCTGTAGACAAGGCTG-3’  5’-AAGTCTGCTCGGGTCATCAAC-3’  5’-GAGCCCGTGTATCTGCTGG-3’  5’-GCCGAGTGGAAACTTTTGTCG-3’  5’-GGCAGCGTGTACTTATCCTTCT-3’  5’-CTTGAATCCCGAATGGAAAGGG-3’  5’-GTGTATATCCCAGGGTGATCCTC-3’  5’-TTTGTGGGCCTGAAGAAAACT-3’  5’-AGGGCTGTCCTGAATAAGCAG-3’  5’-GGCTCCTGGCAAAAGGTCA-3’  5’-CTGCGTAGTTGTGCTGATGT-3’  5’-CCCACATGAAGCGACTTCCC-3’  5’-CAGGTCCAGGAGATCGTTGAA-3’ |
| *SNORD88B (Forward)* | 5’-GACCCCGTGATGTCCAGC-3’ |
| *SNORD88B (Reverse)* | 5’-TCAGAACCCCGGATGTCAAAG-3’ |
| *SNORA74A (Forward)* | 5’-CGGTTGTCAGCTATCCAGGC-3’ |
| *SNORA74A (Reverse)* | 5’-CACTCTCAGAGGTGCAAAACACTG-3’ |
| *SNORA6 (Forward)* | 5’-TGCACACTATTAAAGCTCAGGGTGG-3’ |
| *SNORA6 (Reverse)* | 5’-CTATGTGGCAGCGAGTACTACCA-3’ |
| *SNORA62 (Forward)* | 5’-GGAGTTGAGGCTACTGACTGG-3’ |
| *SNORA62 (Reverse)* | 5’-AGCGAAAACTTGCCCCTCAT-3’ |
| *SNORA74B (Forward)* | 5’-TTGTCTTCATCCGGTTGCCT-3’ |
| *SNORA74B (Reverse)* | 5’-CTTAGCACACTCTCGGGGAA-3’ |
| *SNORA74C (Forward)* | 5’-AGCAGTAGTCAGCTGTCTGGA-3’ |
| *SNORA74C (Reverse)* | 5’-TGATGGACGCAACTGGATGA-3’ |
| *SNORA74D (Forward)* | 5’-GGAGAACAGCAGCCAATAGC-3’ |
| *SNORA74D (Reverse)* | 5’-GCATCCTGGGGTAGTCAACA-3’ |
| *SNORA54 (Forward)* | 5’-ACTGACTGACTGGTGGCTTTCAG-3’ |
| *SNORA54 (Reverse)* | 5’-TGAGTCCAAAGGATCTCAG-3’ |

**Table S5. ASOs against *SNORA74A* used in this study**

| ASOs | ASO sequences |
| --- | --- |
| ASO-h  ASO | mCmCmAmAmAC∗C∗T∗C∗A∗A∗T∗A∗A∗A∗mGmAmCmAmG  mGmCmCmGmAC∗C∗C∗T∗T∗A∗G∗T∗A∗A∗mGmGmCmAmG |

**Table S6. Primers for ChIP assay in this study**

| Genes | Sequences |
| --- | --- |
| *-400~-200 (Forward)* | 5’-CCCGGTCCGACTTTTAGGTTTG-3’ |
| *-400~-200 (Reverse)* | 5’-CCCCCAGTACACACCCAGAG-3’ |
| *-700~-500 (Forward)* | 5’-GAGACAGGGTCTTACTCTGTTGC-3’ |
| *-700~-500 (Reverse)* | 5’-GACCAGCCTGGCCAACATGGT-3’ |
| *-1000~-800 (Forward)* | 5’-AGGGACACTGCACTCCAGC-3’ |
| *-1000~-800 (Reverse)* | 5’-TGCTCTCAGCCCTCCCAG-3’ |
| *-1300~-1100 (Forward)* | 5’-CAGGAGAATGGTGTGAACCCAGG-3’ |
| *-1300~-1100 (Reverse)* | 5’-ACCCCCACAATCTGCCAAGTAG-3’ |
| *-1600~-1400 (Forward)* | 5’-AAAGGCAATTGATCAAGGCTGGG-3’ |
| *-1600~-1400 (Reverse)* | 5’-GCCTCAGCCTCCCAAAGTGTTG-3’ |
| *-1900~-1700 (Forward)* | 5’-ATACAGGGCTGGAGCCTTAGC-3’ |
| *-1900~-1700 (Reverse)* | 5’-GGAGGAACTTCCAGTCTGGGG-3’ |
| *-2200~-2000 (Forward)* | 5’-AGCCATACCTGCCTTGTAGGTG-3’ |
| *-2200~-2000 (Reverse)* | 5’-ATCCCAGCAGAGGGACTGGC-3’ |
| *-2500~-2300 (Forward)* | 5’-AAGAGTTTGAGACTAGCCTAGCCTACA-3’ |
| *-2500~-2300 (Reverse)* | 5’-AAAGTCCCTCTGTCACCCAGAC-3’ |
| *-2800~-2600 (Forward)* | 5’-GACCAGCCTGACCAACATGGAGAAACT-3’ |
| *-2800~-2600 (Reverse)* | 5’-GAGATGGAGTTTTGCTCTTGTTGCCCC-3’ |
| *-3100~-2900 (Forward)* | 5’-CAGCCTGGACAACATGGTGAAACCCT-3’ |
| *-3100~-2900 (Reverse)* | 5’-AAGTCTCGGTCTGTGGCCCAGG-3’ |

**Table S7. Clinical characteristics of HCC patients.**

| **Characteristics** | **Hepatocellular carcinoma**  **(n=5) *** |
| --- | --- |
| Sex |  |
| Male | 3 (60%) |
| Female | 2 (40%) |
| Age (yr) |  |
| >55 | 5 (100%) |
| HBV positive | 5 (100%) |
| AFP (ug/L) |  |
| ≤20 | 2 (40%) |
| >20 | 3 (60%) |
| Tumor size (cm) |  |
| ≤5 | 3 (60%) |
| >5 | 2 (40%) |
| Cirrhosis |  |
| With | 5 (100%) |
| Differentiation |  |
| Low | 3 (60%) |
| Medium | 1 (20%) |
| High | 1 (20%) |
| * Data are shown as numbers (%). | |
